# Revealing an Intercalation Nature of High‐Capacity Conversion Cathode Materials for Fluoride‐Ion Batteries by Operando Studies

**DOI:** 10.1002/smtd.202500374

**Published:** 2025-06-04

**Authors:** Hong Chen, Roland Schoch, Jean‐Noel Chotard, Yannick M. Thiebes, Kerstin Wissel, Rainer Niewa, Matthias Bauer, Oliver Clemens

**Affiliations:** ^1^ University of Stuttgart Institute for Materials Science Materials Synthesis Group Heisenbergstraße 3 70569 Stuttgart Germany; ^2^ Paderborn University Institute for Inorganic Chemistry and Center for Sustainable Systems Design (CSSD) Warburger Str. 100 33098 Paderborn Germany; ^3^ Université de Picardie Jules Verne Laboratoire de Réactivité et de Chimie des Solides CNRS‐UMR 7314 Amiens 80039 France; ^4^ University of Stuttgart Institute for Inorganic Chemistry Pfaffenwaldring 55 70569 Stuttgart Germany

**Keywords:** all solid‐state fluoride ion batteries, conversion–based cathode materials, operando X‐ray absorption spectroscopy, operando X‐ray diffraction, structure evolution

## Abstract

To improve the performance of high‐energy‐density electrode materials for all‐solid‐state fluoride‐ion batteries (ASSFIBs), it is important to understand the structure and phase evolution during operation, which is closely correlated to capacity fading. In this study, an *operando* cell is designed compatible with laboratory X‐ray diffraction (XRD) to monitor real‐time structural changes of bismuth trifluoride (BiF_3_) cathodes and degradation of the ionic conductor BaSnF_4_ under negative potentials at 100 °C. Supported by ex‐situ XRD, our results reveal a multi‐step defluorination of BiF_3_: from orthorhombic (o‐BiF_3_) to cubic (c‐BiF_3_), then to distorted orthorhombic (o’‐BiF_3_), and finally to metallic bismuth (Bi), indicating partial intercalation‐type character. Formation of bismuth oxidefluoride (BiOF) beyond 200 mAh g^−1^ is attributed to oxygen impurities introduced via solid‐state synthesis. *operando* X‐ray absorption spectroscopy (XAS) confirms a continuous reduction of Bi_3+_ to Bi_0_ with intermediate phases. Rietveld refinement quantifies the phase fractions and structural transitions, enabling a model for BiF_3_ defluorination. Comparison of *operando* XRD and XAS reveals that BaSnF_4_ contributes transport of both fluoride and oxygen impurities, leading to BiOF formation. BaSnF_4_ also exhibits a broad stability window, with degradation occurring below −200 mV, rather than the expected −50 mV vs. Sn/SnF_2_.

## Graphical abstract

This study applies operando XRD & XAS at 100 °C to monitor structural evolution in BiF_3_ cathodes and electrolyte BaSnF_4_ in all solid‐state fluoride ion batteries. It reveals multi‐step BiF_3_ defluorination, oxygen impurity‐induced BiOF formation, and a wide stability window for BaSnF_4_. The findings underscore BaSnF_4_´s crucial role in O^2‐^ and F^‐^ transport, linking the synthesis method on cell performance.

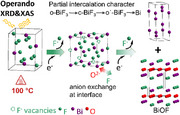

## 1 Introduction

With increasing demand of high‐capacity, safe, rechargeable energy storage systems for efficient use of fossil and renewable energy, the development of alternative battery systems has been strongly motivated.^[^ ^1^, ^2^, ^3^, ^4^, ^5^ ^]^ The concept of battery systems based on anion transport was introduced more than half‐century ago,^[^ ^6^, ^7^ ^]^ and the fluoride ion, as the most chemically stable shuttle anion, has been widely investigated for use in fluoride‐ion batteries (FIBs).^[^ ^8^ ^]^ Recent demonstrations of high‐energy‐density rechargeable FIBs have significantly boosted interest in this field.^[^ ^9^, ^10^, ^11^ ^]^ Most research has focused on improving the conductivity of electrolytes at room temperature as well as developing high‐performance electrode materials.^[^ ^12^, ^13^, ^14^, ^15^, ^16^, ^17^ ^]^

Metal fluorides (such as BiF_3_, CuF_2,_ and CeF_3_) are commonly suggested as cathode active materials (CAMs) in many battery systems because of their high specific capacity.^[^ ^15^, ^18^, ^19^ ^]^ Despite the common concern that large volume changes of CAMs observed for conversion‐based phase transformation (recrystallization) lead to loss of contact and poor capacity retention upon cycling, the detailed phase transformations occurring upon charge/discharge processes are only little understood. In the case of BiF_3_, previous investigations on reaction mechanism were mostly carried out on LIBs (BiF_3_ + 3 Li^+^ + 3 e^‐^ → 3 LiF + Bi) and/or FIBs (BiF_3_ + 3 e^‐^ → Bi + 3 F^‐^) based on liquid electrolytes.^[^ ^20^, ^21^, ^22^, ^23^, ^24^, ^25^ ^]^ Konishi's group proposed that the poor cycling performance of BiF_3_ electrodes in Lithium‐ion batteries (LIBs) could be attributed to insufficient discharge reactions as well as the production of Bi‐metal and organic electrolyte impeding surface charge reactions.^[^ ^22^ ^]^ Yamanaka's group observed different reactivity and defluorination mechanisms of different BiF_3_ modifications (orthorhombic structure (o‐BiF_3_) and cubic structure (c‐BiF_3_)) in FIBs with liquid electrolytes by in situ Raman microscopy.^[^ ^23^, ^24^ ^]^ They proposed that the defluorination of o‐BiF_3_ occurred via a direct defluorination mechanism while the defluorination of c‐BiF_3_ was most likely controlled by the competition between a direct mechanism and a dissolution‐deposition mechanism. Li and his group^[^ ^25^ ^]^ presented an aqueous dual‐ion battery in which the pre‐addition of Bi_7_F_11_O_5_ to the BiF_3_ electrode composite can enhance the structural stability and reversibility of Bi‐F‐O phases by formation of intermediate phase Bi_7_F_11_O_5_ during defluorination, which can reduce the volume changes and improve electronic conductivity.^[^ ^25^ ^]^ However, there is a poor understanding of the evolution of the BiF_3_/Bi electrode in all‐solid‐state fluoride ion batteries, both theoretically and experimentally. Rongeat et al.^[^ ^19^ ^]^ reported the formation of an oxidefluoride phase, referred to as BiF_3‐2x_O_x_ (*Fm*‐3*m*, 0.41 ≤ x ≤ 0.52), during the fluorination of Bi_2_O_3_ (19 wt.% detected by X‐ray diffraction (XRD)) present in the commercially purchased Bi powder. Based on their X‐ray diffraction analysis, they suggested that BiF_3‐2x_O_x_ (0.41 ≤ x ≤ 0.52) cannot be defluorinated within subsequent discharging. They noted that a more detailed data analysis was hindered due to intense and broad reflections from the used electrolyte phase La_1‐x_Ba_x_F_3‐x_ (0.05 ≤ x ≤ 0.15, denoted as LBF), which overlapped with signals from the Bi‐based phases. Later in 2017, Grenier et al.^[^ ^26^ ^]^ studied the involvement of various bismuth oxidefluoride phases in electrochemical reactions using symmetric cells (BiF_3_/Bi – LBF – Carbon) via synchrotron‐based XRD and pair distribution function (PDF) analysis. The CAMs consisted of 24 wt.% BiO_0.55_F_1.9_ (*R*‐3*m*) and Bi_2_O_3_ (relative 29–44 wt.%, *P*2_1_/*c*). To date, it has been observed that electrochemical reactions within BiF_3_/Bi active materials can involve multiple species and phases depending sensitively on different electrolytes and cell testing conditions, however, these reactions have only been clarified in part. Moreover, rechargeable all‐solid‐state FIBs (ASSFIBs) with novel room‐temperature solid electrolytes, such as BaSnF_4_, PdSnF_4_^[^ ^18^ ^]^ and recently CsPb_0.9_K_0.1_F_2.9_^[^ ^27^ ^]^ have been reported. The electrochemical compatibility of BiF_3_/Bi as CAMs with those electrolytes has been successfully demonstrated at lower temperatures. However, a deeper understanding of the involved phases and their structure evolution is still required. Thus, there are many open questions that remain to be addressed to improve the cyclability and stability of ASSFIBs using conversion‐based electrode materials. These include stability with the electrolyte, phase transformations, and the maintenance of active interfaces within CAM particles. Such experimental studies are typically time‐consuming, and time‐efficient in situ/*operando* analyses, widely applied in the field of lithium‐ion batteries at room/moderate temperatures,^[^ ^28^, ^29^, ^30^, ^31^, ^32^ ^]^ have not yet been reported for fluoride ion batteries. Further, since these cells are operated at elevated temperature, ex situ studies at room temperature may not accurately represent the behavior of the materials at operation conditions.

Achieving reliable *operando* elevated temperature measurements requires stable, homogeneous heating, robust cell sealing, and preserved surface contact. Therefore, the cell design and measurement setup must be carefully adjusted and optimized accordingly. Though *operando* XRD/XAS measurements under electrochemical conditions at high temperatures have been demonstrated in solid oxide fuel cell (SOFC) research,^[^ ^33^, ^34^ ^]^ the associated challenges and cell configuration differ from those of all‐solid‐state batteries (ASSBs). To the best of our knowledge, there are no prior reports of *operando* XRD and XAS measurements conducted at elevated temperatures (e.g., 100 °C) specially for all‐solid‐state batteries.

In this paper, we thus demonstrate an *operando* study of the first discharge process of the cell BiF_3_|BaSnF_4_|Sn over a wide potential range at 100 °C with X‐ray diffraction (XRD) and X‐ray absorption spectroscopy (XAS), respectively. For the first time, we reveal that the phase transition of the initial orthorhombic phase of BiF_3_ (o‐BiF_3_) into metallic Bi is not a direct reduction process. In contrast to a simple reconstructive conversion reaction, three intermediate phases are identified during this process. *operando* XRD measurements provide real‐time monitoring of the structure evolution of the BiF_3_ composite, correlating the transition process to the corresponding cell potential (capacity). This approach differs from ex situ analysis and even reveals the formation of an intermediate orthorhombic Bi‐based phase, which has not been reported so far. This allows for the deduction of a reaction mechanism, providing new insights into the degradation of the electrolyte material and overall cell performance, which will contribute to further improving the cycling stability of high‐capacity BiF_3_ cathode material while reducing the operating temperature closer to ambient conditions.

## 2 Results and Discussion

### 2.1 Structure and Characterization of BaSnF_4_ and BiF_3_ Cathode Material

**Figure 1:**
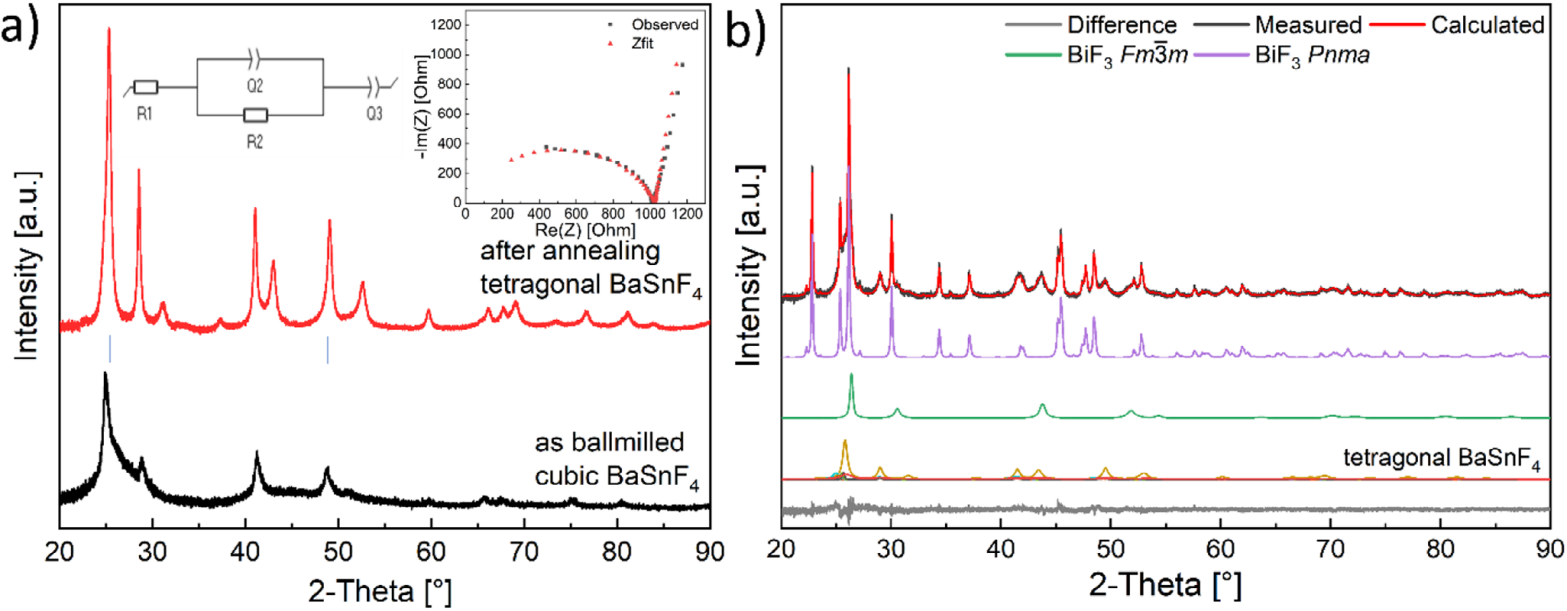
a) XRD patterns of BaSnF_4_ after ball milling and after the annealing process (blue vertical lines point to the presence of Ba‐rich t‐BaSnF_4_ due to the partial incorporation of SnF_2_ into cubic BaF_2_), inset is the Nyquist plot of t‐BaSnF_4_ measured at 25 °C; b) refined XRD pattern of BiF_3_ composite.

The crystal structure and temperature dependence of the ionic conductivity of tetragonal‐BaSnF_4_ (referred to as t‐BaSnF_4_) synthesized in this work were evaluated by XRD and EIS. The EIS spectra of t‐BaSnF_4_ (inset) as well as XRD patterns of BaSnF_4_ after applying the milling procedure described in the methods section and after subsequent annealing are presented in **Figure** **1**a. After applying the ball milling procedure, the sample mainly consists of nanocrystalline cubic‐BaSnF_4_, along with phase fractions closer to the starting materials SnF_2_ and BaF_2_ with certain amounts of defects possibly introduced on milling (indicated by small changes of lattice parameters) due to the mechanical impacts. The broadening and asymmetry (extended tails to higher diffraction angles) for the reflections can be attributed to inhomogeneous compositions (and resulting deviations in unit cell dimensions) of SnF_2_ and BaF_2_ according to Ba_0.5±x_Sn_0.5_∓_x_F_2_ as well as amorphization of incorporated pristine materials. From Rietveld analysis, we obtained a cell parameter of *a* = 6.2081(4) Å for cubic‐BaSnF_4_, which agrees with the value of 6.194(1) Å reported previously^[^ ^35^ ^]^ and also suggests the possibility of local deviation of cation composition from an ideal Ba_0.5±x_Sn_0.5_∓_x_F_2_ with x = 0. Similarly, Mercadier et al.^[^ ^36^ ^]^ recently studied the deviations in the local cationic composition of cubic‐BaSnF_4_ by total‐scattering PDF and AIMD simulations data and reported the subsequent highly inhomogeneous fluorine substructure.

The initial cubic phase obtained after milling was observed to transform into a tetragonal structure following an annealing process, as shown in Figure 1a (red pattern). The sharp reflections indicate good crystallinity of the annealed sample. The ionic conductivity of the annealed BaSnF_4_ calculated from the fitted total resistance in the fitted equivalent circuit is 3.5 × 10^−4^ S cm^−1^ at 25 °C. Both phase structure and ionic conductivity of the annealed t‐BaSnF_4_ are consistent with literature values for t‐BaSnF_4_.^[^ ^18^, ^37^ ^]^ In Figure 1a, the asymmetry of certain reflections (marked by blue vertical lines) at lower angles can be easily recognized. This asymmetric characteristic has been rarely noticed,^[^ ^35^, ^37^ ^]^ where it was attributed to the remaining incorporated cubic‐BaF_2_ and X‐ray amorphous SnF_2_ or cubic‐BaSnF_4_. However, simply adding starting materials and cubic‐BaSnF_4_ into the structure analysis gives a poor fit in our case. The reflections, which show the asymmetric tail at lower 2‐theta values, are correlated to lattice planes that have a strong dependence on the *c* lattice parameter in a tetragonal unit cell, for example, reflections at ≈25.8°, 43.4°, 49.4° and 52.8° corresponding to (102), (114), (212) and (106). In contrast, (h k 0) reflections do not show such asymmetric characteristics. Considering the deviation from the ideal cationic composition in cubic‐BaSnF_4_ and the larger ionic radius of Ba^2+^ as compared to Sn^2+^,^[^ ^38^ ^]^ we used a model with inhomogeneous distributions of Ba and Sn on the cation sites in the formed t‐BaSnF_4_ phase after annealing, where the occupancy of Ba at the Sn1 site can lead to an expansion along the *c*‐axis within the unit cell. The corresponding Rietveld refinement shows that the synthesized t‐BaSnF_4_ has partial mixed occupancy of Ba and Sn at the Sn1 site, which can further be different for various grains according to Ba_1±x_Sn_1_∓_x_F_4_ (from the ideal structure of t‐BaSnF_4_^[^ ^35^ ^]^). By taking such a distribution of t‐BaSnF_4_ phases with different Ba/Sn occupancies at Ba1 and Sn1 sites into account, a good fit can be realized (a detailed fitting model is given in Figure S2, Supporting Information). It is observed that repeated processes of milling and annealing can improve the homogeneity of Ba and Sn incorporation (see Figure S3, Supporting Information). However, full homogeneity in the distribution of Sn and Ba cannot be achieved by the suggested synthesis process, even after three repetitions; likewise, extending the annealing time or increasing the annealing temperature cannot relieve the asymmetry of reflections. Nevertheless, the functionality of the electrolyte phase is not influenced, and the conductivity is sufficiently high for both room temperature and elevated temperature operation of FIBs.

The XRD pattern of the as‐prepared BiF_3_ cathode composite containing t‐BaSnF_4_ and carbon nanofibers is shown in Figure 1b. The as‐received BiF_3_ powder consists of two modifications, orthorhombic (space group *Pnma*, ≈85 wt.%) and cubic (space group *Fm*‐3*m*, ≈15 wt.%), see Figure S4 and Table S1 (Supporting Information). The lattice parameter *a* in cubic BiF_3_ in pristine powder is refined to be 5.8501(3) Å, in good agreement with the previously reported value (5.865 ± 0.06 Å at room temperature^[^ ^39^, ^40^ ^]^), and the corresponding reflections show a broad characteristic. Considering the rather large error of literature value we assumed that the cubic modification in the as‐received BiF_3_ powder may not have the ideal composition of BiF_3_. We hypothesized a compositional fluctuation due to a combination of vacancies on fluorine sites and oxygen incorporation. This is consistent with previous reports on Bismuth oxidefluoride denoted as BiF_3‐2x_O_x_ (0.41 ≤ x ≤ 0.52), which also crystallizes in the cubic space group *Fm*‐3*m*. For x = 0.51,^[^ ^26^, ^41^ ^]^ the lattice parameter *a* is ≈5.85 Å and it increases with increasing fluorine content. To verify this hypothesis, elemental analysis of the BiF_3_ powder was conducted, revealing that the purchased BiF_3_ has an oxygen content of 0.78(37) wt.%. This indicates that the cubic BiF_3_ is primarily a defect BiF_3‐δ_ accompanied by small amounts of oxygen impurities. Furthermore, apart from t‐BaSnF_4_ and BiF_3_ with two modifications identified, no other additional phases were observed in the as‐prepared cathode composite. In addition, we also performed an elemental analysis of the as‐prepared BiF_3_ cathode composite (see Table S2, Supporting Information) as well as the individual other ingredients (t‐BaSnF_4_, CNF as well as the pristine materials for electrolyte preparation). The analysis revealed an overall oxygen content in the cathode composite of 1.40(17) wt.%, which mainly originates from the solid electrolyte (1.08(4) wt.% of oxygen content measured). The increased oxygen content in the cathode composite likely results from the electrolyte and carbon nanofibers during the milling process, suggesting that additional oxygen species (O^2‐^) are present in the phases in contact with the CAM BiF_3_.

### 2.2 Structure Evolution of Bi/BiF_3_ Studied by Ex Situ X‐Ray Diffraction

As is common for studies of electrode materials for fluoride ion batteries, the temperature stability of the BiF_3_ composite was examined by heating the composite to the cell's operating temperature in an argon atmosphere for ≈50 and ≈75 h (which corresponds to a typical cell operation time). It is indicated that the composite exhibits good thermal stability (see Figure S5 and Table S4, Supporting Information for a comparison of diffraction patterns as well as structure information before and after heating). The observed changes are minor and fall within the margin of error as determined by Rietveld analysis.

**Figure 2:**
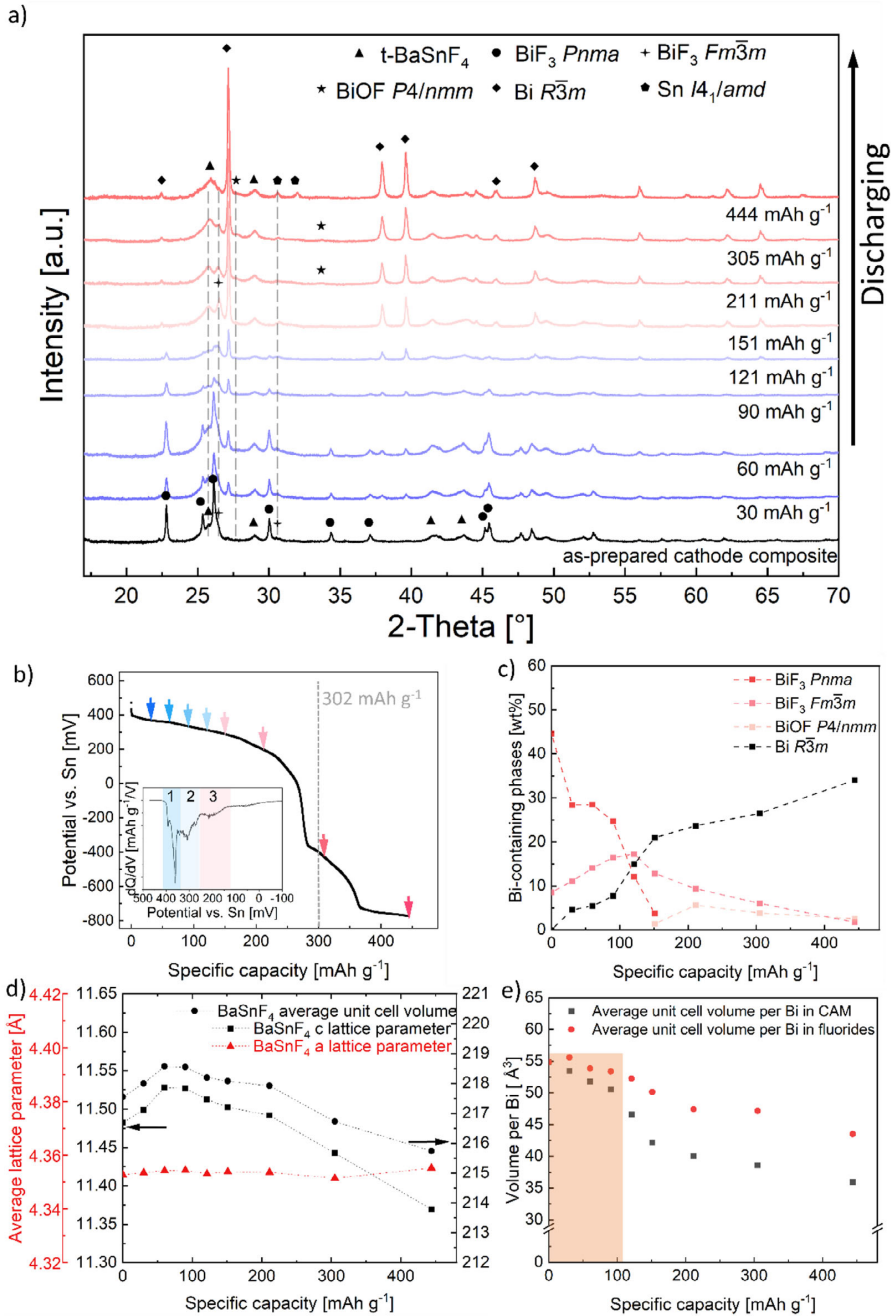
a) Series of XRD patterns recorded at room temperature on different cell pellets at different discharge states. Reflections attributed to different phases are marked with symbols. b) Typical potential curve of cell pellets discharged in a Swagelok cell at 100 °C with 20 uA cm^−2^ current density for ex situ XRD investigation. Colored arrows refer to the specific capacity values that have been investigated in this work. Inset is the dQ/dV plot of the potential curve in the potential range from 500 to −100 mV. c) Weight fraction evolution of Bi‐containing phases evaluated by Rietveld analysis plotted against discharge capacity. d) Lattice parameters and average unit cell volume evolution of t‐BaSnF_4_ evaluated by Rietveld analysis plotted against discharge capacity. e) The average unit cell volume per Bi atom in Bi‐containing phases as well as in bismuth fluoride phases plotted against discharge capacity.

A potential curve against capacity during first discharging to −775 mV versus Sn/SnF_2_ of the cell BiF_3_|BaSnF_4_|Sn is shown in **Figure** **2**b. Based on the changing tendency (slope) of the decreasing potential with capacity and corresponding dQ/dV plot (inset), the first defluorination of BiF_3_ cathode is primarily composed of three steps for reaching the theoretical capacity of BiF_3_ (302 mAh g^−1^). In order to reveal the structural evolution of CAM BiF_3_ as well as the structural stability of the electrolyte, a series of ex situ X‐ray diffraction patterns (Figure 2a) was recorded on BiF_3_|BaSnF_4_|Sn pellets after discharging to specific capacities from 30 to 444 mAh g.^−1^ The specific capacities investigated by ex situ XRD are marked in Figure 2b with colored arrows. The XRD pattern of the as‐prepared cathode composite (black curve) serves as a reference, with the initial phases discussed in the previous section.

Upon discharging, the defluorination of BiF_3_ toward Bi and the structural stability of BaSnF_4_ are evidenced by changes in the intensity and positions of reflections of corresponding phases. As shown in Figure 2a, with ongoing defluorination, the reflections corresponding to orthorhombic BiF_3_ (at angles of 22.80°, 25.36°, 26.18° and 30.03° for (101), (020), (111) and (210), respectively) decrease in intensity, while reflections corresponding to metallic Bi emerge. Simultaneously, the reflections at angles of 26.35° and 30.52° identified to (111), (200) of cubic BiF_3_ increase in intensity until a capacity of 120 mAh g^−1^; beyond this capacity, their intensities decrease and the reflections disappear completely at 444 mAh g^−1^. Furthermore, starting at a capacity of 150 mAh g^−1^ weak reflections at angles of 27.72° and 33.73° appear. These weak reflections can be assigned to tetragonal BiOF (space group *P*4*/nmm* ^[^ ^42^ ^]^), and they persist with only slightly reductions in intensity even as the cell potential decreases to −775 mV. Throughout the defluorination, the reflections attributed to metallic Bi (and thus the corresponding weight fraction) increase continuously, with their strongest increase per discharge capacity at a specific capacity of ≈120 mAh g^−1^; a numerical derivative of the weight fraction versus capacity gives a slope ≈0.64 wt.% g (mAh)^−1^ at 120 mAh g,^1^ which is far larger compared to what would be expected for the direct defluorination of o‐BiF_3_ to form fully crystalline metallic Bi (100 wt.%/302 mAh g^−1^ = 0.33 wt‐% g (mAh)^−1^). Thus, the observed behavior of the reflections from different phases can only be explained if o‐BiF_3_ does not transform directly to metallic Bi on the defluorination process, but that the defluorination is happening via a most likely defect‐rich cubic intermediate phase with composition BiF_3‐x_, which is chemically different to the cubic phase initially present in the BiF_3_ composite (where the high symmetry is induced from the combination of oxygen incorporation and fluoride vacancies). This is contrary to a straight reconstructive transformation model between the most stable modification of BiF_3_ (*Pnma*) and metallic Bi (*R*‐3*m*).^[^ ^20^, ^23^ ^]^

In Figure 2c, the relative weight fractions of Bi‐containing phases, evaluated by Rietveld refinement, are plotted as a function of specific capacity. The evolution of the weight fractions on specific capacity can be described as follows: During the initial discharge process, the disappearance of orthorhombic BiF_3_ is accompanied by the formation of the intermediate cubic BiF_3‐x_ up to a capacity of 120 mAh g^−1^, while the content of metallic Bi, though formed to a small extent initially, remains constant. At capacities exceeding 150 mAh g^−1^, a tetragonal bismuth oxidefluoride phase (BiOF) is identified, with a weight fraction of ≈5 wt.%. The formation of BiOF has been observed during the electrochemical fluorination process of Bi particles in the presence of Bi_2_O_3_ (≈29 to 44 wt.%),^[^ ^26^ ^]^ and it has been reported that oxide presence significantly affects the behavior of fluoride conversion electrodes. In the previous section, the elemental analysis reveals that in our case the marginal oxygen content (≈1.40(17) wt.%) in BiF_3_ cathode composites primarily originates from electrolyte during composite preparation and is distributed among particles of different phases, suggesting the correlation between the formation of BiOF and the oxygen impurity. The cell sealing integrity, confirmed by the stable ionic conductivity of moisture‐sensitive t‐BaSnF_4_ at the cell operating temperature, allows us to reasonably rule out moisture absorption from the air as an external oxygen source during measurement and the electrolyte oxygen impurities remain as the only possible internal oxygen source in the system (see Figure S16, Supporting Information and accompanying comments). The analysis of lattice parameters of t‐BaSnF_4_ (shown in Figure 2d, discussed in the last paragraph of this section) indicates that, though it has not been reported that t‐BaSnF_4_ is capable of conducting oxygen anions, O^2‐^ ions diffusion along solid‐solid interfaces and O‐F exchange between electrolyte and BiF_3_ electrode material are considered possible. This will give the possibility of O^2‐^ agglomeration at interfaces during defluorination. After discharging to a certain capacity (120 mAh g^−1^ in our case), the reduced Bi/F composition and the O^2‐^ agglomeration promote the formation of thermodynamically more stable bismuth oxidefluoride. The mechanism underlying BiOF formation will be discussed in detail later, along with additional in situ analysis. However, this phase does not disappear at higher discharge states under the formation of more metallic Bi, which could be assigned to the strong O─Bi bond, relatively poor O^2‐^ conductivity of composite, or the accessibility loss from electron/F^‐^ conducting particles due to the volume change. Though a direct reduction from BiF_3_ to Bi metal cannot be entirely ruled out from ex situ results, the phase transformation from o‐BiF_3_ to c‐BiF_3_ prior to the significant increase in Bi content is clearly revealed. The correlation between the two structures of BiF_3_ will be further discussed along with in situ results.

Since the Bi‐containing phases carry different volumes per Bi (Table S5, Supporting Information), i.e., the electrode undergoes a volume change when the state of charge evolves, the degree of volume change can be represented by averaging the unit cell volume per Bi atom in bismuth fluorides and overall Bi‐containing phases (Figure 2e). For a direct conversion of BiF_3_ to Bi, an almost linear (detailed curve see Figure S8, Supporting Information) dependency between Bi weight fraction and specific capacity would be expected for an exclusive conversion of BiF_3_ to Bi with full crystallinity. However, within a certain capacity range different slopes were found, correlating to different intermediate phases, e.g. where intermediate cubic BiF_3_ phase forms (marked in orange color), the overall volume changes significantly slowly. Since volume changes are factors influencing the cycling stability, determining the capacity ranges with low volume changes can help to improve the cycling stability of conversion systems.^[^ ^43^, ^44^ ^]^

Figure 2d shows the weight‐averaged *c* lattice parameter and volume per unit cell of t‐BaSnF_4_ in the cathode composite as a function of discharge capacity. It is revealed that during the discharge process t‐BaSnF_4_ undergoes a cell relaxation mostly along the *c* direction resulting in an increase and then a decrease of unit cell volume until 250 mAh g^−1^, ≈217 Å,^[^ ^3^ ^]^ which could be attributed to defects (anion vacancy) introduced and eliminated by F^−1^ diffusion through the solid electrolyte as well as possible O‐F‐exchange at grain boundaries of the electrolyte and/or with the BiF_3_ electrode material. Based on the detailed XAS measurements discussed in the last section, the lattice parameter changes observed on t‐BaSnF_4_ are not redox‐mediated, but likely originate from the O‐F‐exchange with the BiF_3_ electrode material. This would also well explain the overall formation of BiOF, though the initial BiF_3_ phases contain less oxygen than what could contribute to the amount of BiOF observed on the state close to full discharge. At capacity exceeding the theoretical capacity of BiF_3_ (302 mAh g^−1^), the decrease in the *c* lattice parameter is most likely attributed to the decomposition of the solid electrolyte. At a capacity of 444 mAh g^−1^, two reflections at 2‐theta angles of 30.61°, 32.0° appear, which can be assigned to the (200) and (101) planes of metallic Sn. This observation suggests that the decomposition of the electrolyte has been induced at potentials below −400 mV. This reduction would likely result in an enrichment of the BaSnF_4_ phase according to Ba_1±x_Sn_1_∓_x_F_4_ or the exsolution of BaF_2_ in an X‐ray amorphous state, which is evidenced by the observation of the rightward shift of the (102) reflection of t‐BaSnF_4_ (from 25.81° to 25.93°) as the capacity increases from 305 to 444 mAh g^−1^. It is surprising that the observed electrochemical stability range of BaSnF_4_ (from 470 to −400 mV against Sn/SnF_2_) is considerably wider than the calculated electrochemical window of BaSnF_4_, reported to be in the order of 450 mV.^[^ ^45^ ^]^ The electrochemical stability window (ESW) of the synthesized BaSnF_4_ determined by the LSV method also indicates a relatively wide stability range (Figure S15, Supporting Information). This could be due to the large overpotential required to break the ordered layer structure nature of BaSnF_4_ though the electrochemical decomposition mechanism behind is not yet clear.

### 2.3 Structure Evolution of Bi/BiF_3_ Studied by *Operando* X‐Ray Diffraction

**Figure 3:**
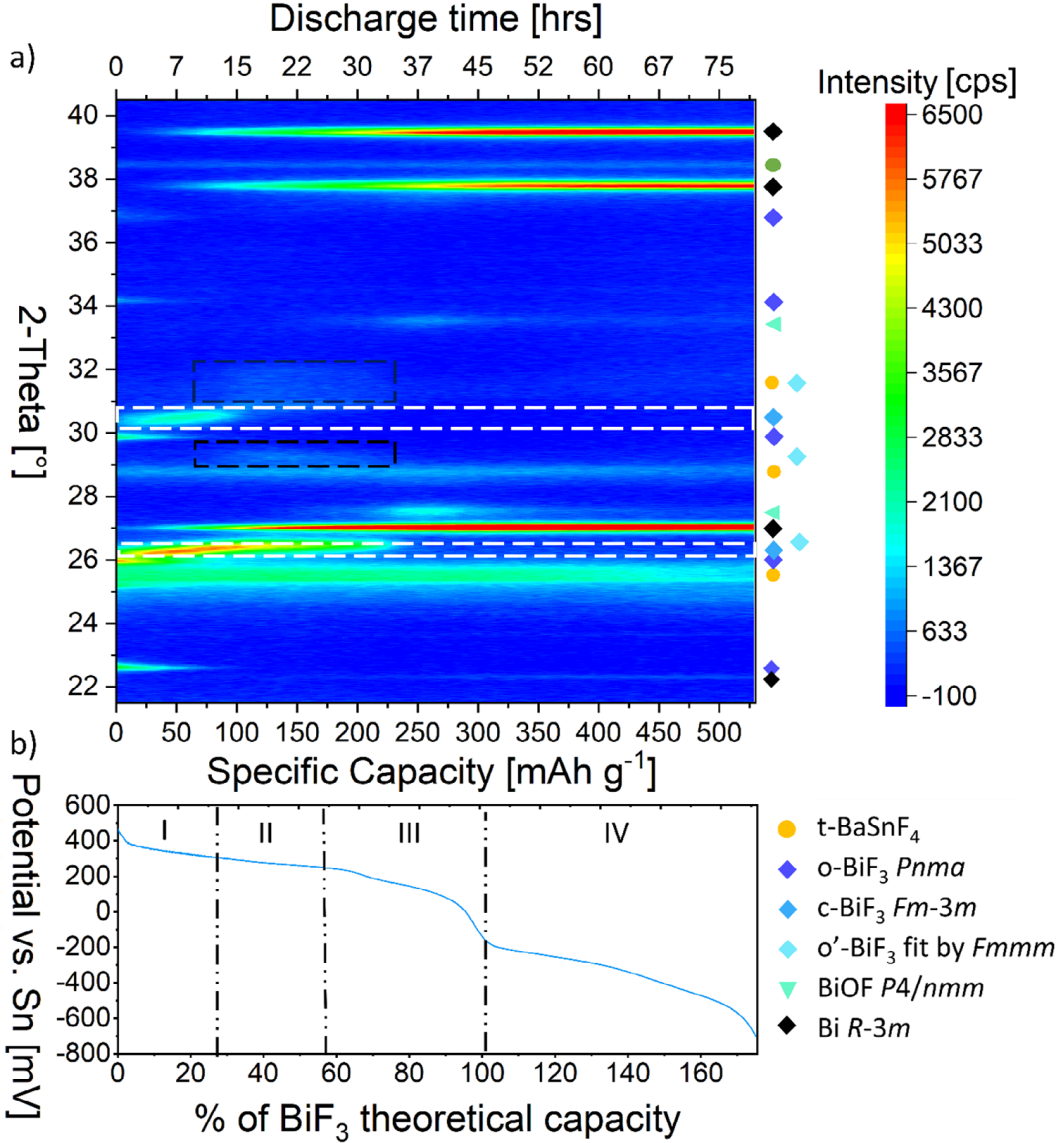
a) 2D‐XRD intensity contour plot of the cell shown in b). The area of white‐dashed rectangles is the evolution of the two dominant reflections (111) & (200) from c‐BiF_3_. b) Cell BiF_3_|BaSnF_4_|Sn potential curve against specific capacity until cut‐off potential −700 mV.

BiF_3_ exists in various modifications depending on temperature and preparation conditions, which have different thermodynamic structure stability at cell operating temperatures (100 °C in this work) and room temperature used for ex situ characterizations. For example, orthorhombic BiF_3_, the thermodynamically most stable phase at room temperature and ambient pressure, can transform into trigonal BiF_3_ (space group *P*‐3*c*1) under high pressure^[^ ^46^, ^47^ ^]^ or after high energy (1100 rpm) ball milling; milder ball milling (600 rpm), as well as soft (350 °C) heat treatment, can recover the orthorhombic phase from the trigonal one.^[^ ^48^ ^]^ Cubic BiF_3_ which has the highest crystal density can be synthesized by precipitating dissolved bismuth nitrate from glycerin by adding a sodium fluoride solution.^[^ ^49^ ^]^ It is also observed when gently (≈120 °C) heating the orthorhombic form under a vacuum.^[^ ^48^ ^]^ Given these structural variations, one should consider that the requirement of cooling the cell and thus the sample for ex situ measurements might induce additional qualitative and quantitative phase changes, which in turn could affect the observed reaction mechanism or result in overseeing metastable intermediate phases involved during cell operation. To gain a comprehensive understanding of the defluorination mechanism of BiF_3_ to Bi under operating conditions and to enhance insights from ex situ XRD into intermediate phases, an *operando* XRD measurement was conducted at 100 °C during discharge. **Figure** **3**b shows the test cell potential curve plot as a function of specific capacity. Figure 3a demonstrates the 2D‐XRD intensity contour plot of the same cell during the discharge process, where each XRD pattern was recorded over a specific capacity interval of ≈7 mAh g^−1^, which is sufficiently low compared to the overall capacity. The intensity variation of the different reflections in the heat map helps to derive the real‐time phase evolution model upon first defluorination of BiF_3_. Selected XRD patterns together with corresponding Rietveld analyses are plotted in Figure S8 (Supporting Information). From these refinements, the individual phases were learned and reasonable limits were derived for scaling parameters, lattice parameters, and reflection widths, which are marked in Figure S8 (Supporting Information) in red color. This helps to generate a stable batch Rietveld analysis in order to extract the state of charge‐dependent details such as lattice parameters and microstructure information.

Though most of the reflections can be assigned to the phases observed in the ex situ study, two remarkable observations are made exclusively in the *operando* analysis and will be described in the following.

First, the formation of another intermediate phase as a result of fluoride ions extraction was observed. We attempted to cool the cell down to further investigate the detailed structure of this phase with complementary methods, such as electron diffraction tomography via the Fast and Automated Diffraction Tomography (Fast‐ADT) technique. However, this phase was found to be metastable at room temperature and could not be detected by ex situ XRD. Its nature was derived from the changes of the XRD patterns described in the following: Beyond a capacity of 75 mAh g^−1^ two shoulders at 2‐theta of 29.2° and 31°–32° appear (marked with dashed black rectangles in Figure 3a). This position is around the (200) reflection of c‐BiF_3_, which decreases in intensity once the additional reflections appear (below 75 mAh g^−1^, it was only observed to shift its position from 30.58° to 30.70° upon defluorination). These additional reflections increase in intensity until a capacity of 125 mAh g^−1^, after which these signals decrease in intensity and they completely disappear at ≈250 mAh g^−1^. Once these new additions decrease and disappear, the formation of t‐BiOF with its indicator reflections at ≈27.7° and ≈33.4° are observed.

Second, it is found that the reflections corresponding to c‐BiF_3_, e.g. the (111) and (200) reflections (marked by white‐dash rectangles in Figure 3a), do not only change their intensity but also show a strong shift to higher diffraction angles (smaller cell volumes). The intensity of c‐BiF_3_ reflections strongly decreases and the phase fully disappears once the metastable phase discussed in the previous paragraphs starts to appear and peaks in intensity respectively.

**Figure 4:**
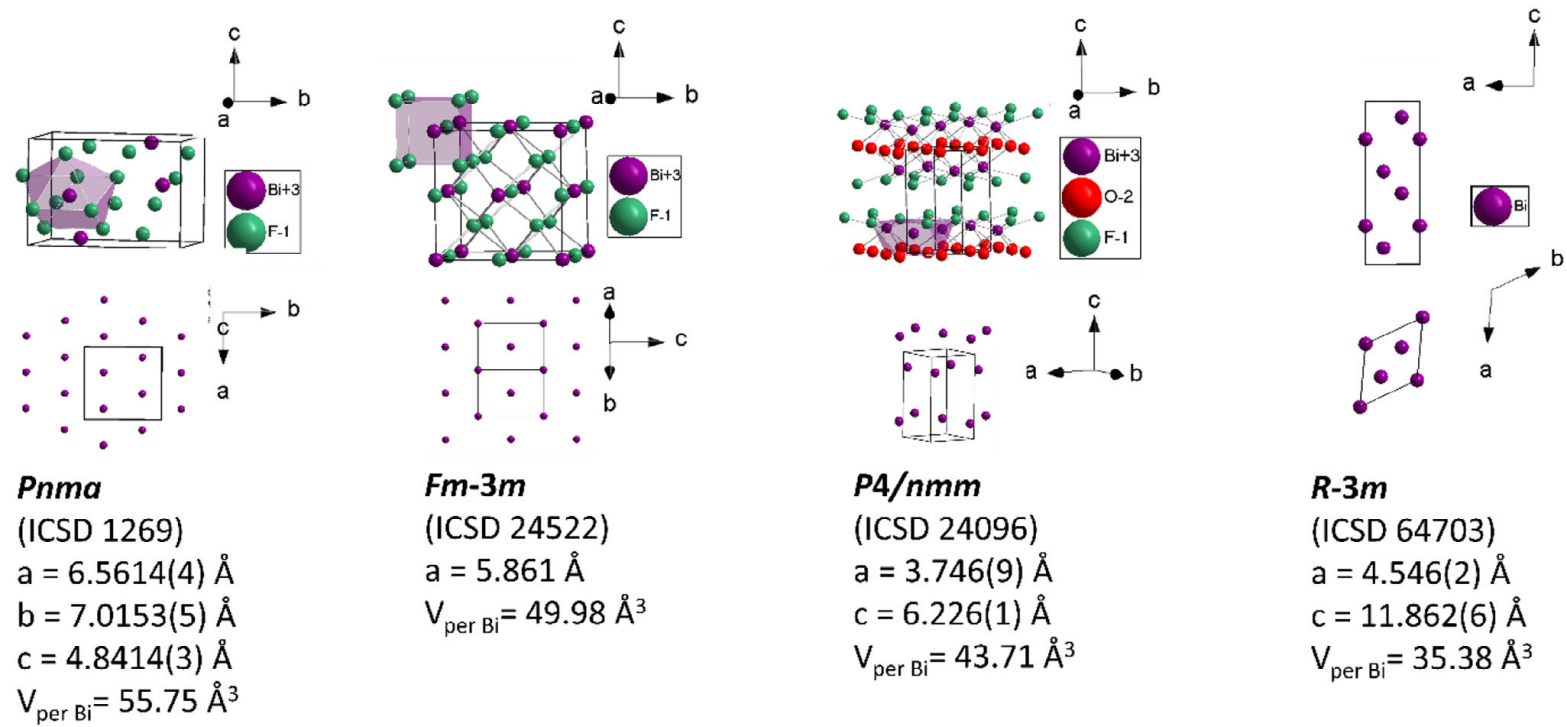
Crystal structure of the two modifications of BiF_3_, tetragonal BiOF (t‐BiOF) as well as metallic Bi and the corresponding Bi‐substructure.

Thus, the intermediate phase is formed from c‐BiF_3,_ and the resulting product from it on increased degree of defluorination is t‐BiOF. Both c‐BiF_3_ and t‐BiOF have Bi‐sublattices which can be described by a regular or distorted F‐centered subcell (**Figure** **4**); since the anions are much weaker scatters than Bi, the diffraction pattern (i.e., relative intensities of main reflections) of all Bi‐O‐F‐phases investigated here are mostly influenced by the bismuth sublattice.

In order to derive structural information on the new intermediate phase, we used these considerations together with the topochemical structural relationship existing between c‐BiF_3_ and BiOF. To derive a structural model, we investigated the intensity distribution around the (200) reflection of the novel intermediate phase in more detail. The integral intensity appearing at lower diffraction angle is approximately half as large as the intensity appearing at higher angles, which seems to be further distributed over a twice as large angular range. Thus, by applying a series of translationengleiche structural transitions, we could set up a structural model of o’‐BiF_3_ in space group *Fmmm* (with a*_Fmmm_* ≈ b*_Fmmm_* ≈ c*_Fmmm_* ≈ a*_Fm_*_‐3_ *_m_*). This results in a splitting of the (200) reflection in three reflections with approximately equal intensity, and gives a good fit of the diffraction patterns (see Figure S8b, Supporting Information, the selected XRD pattern of scan 18 where the partial fit of the broad reflections is plotted); further, such an orthorhombic cell distortion would still result in a non‐splitting of the (111) reflection at 30.7°.

Regarding the electrochemical stability of the electrolyte, it is observed that the most dominant reflection (102) of t‐BaSnF_4_ (2‐theta of 25.78°) starts decreasing in intensity from potential below −250 mV, indicating that the degradation of BaSnF_4_ is taking place. Before that, no changes in the position of reflections from t‐BaSnF_4_ are observed. The potential value for observing electrolyte degradation is ≈100 mV higher than that observed in ex situ results, highlighting that the *operando* study is also allowing for a clearer assignment of the onset of electrolyte decomposition as compared to ex situ studies.

So far, it is revealed clearly by *operando* XRD that the defluorination of BiF_3_ at 100 °C is a complex indirect transition process that involves three intermediate phases with different structure symmetries. Through electrochemical defluorination, BiF_3_ starting with primarily orthorhombic structure undergoes phase a transition to form cubic and then a second orthorhombic structure toward metallic Bi. Bismuth oxidefluoride accumulates the oxygen which was incorporated as an impurity in the BiF_3_ phase and does not disappear on increasing state of discharging anymore (though its phase fraction is reduced beyond ≈250 mAh g^−1^).

**Figure 5:**
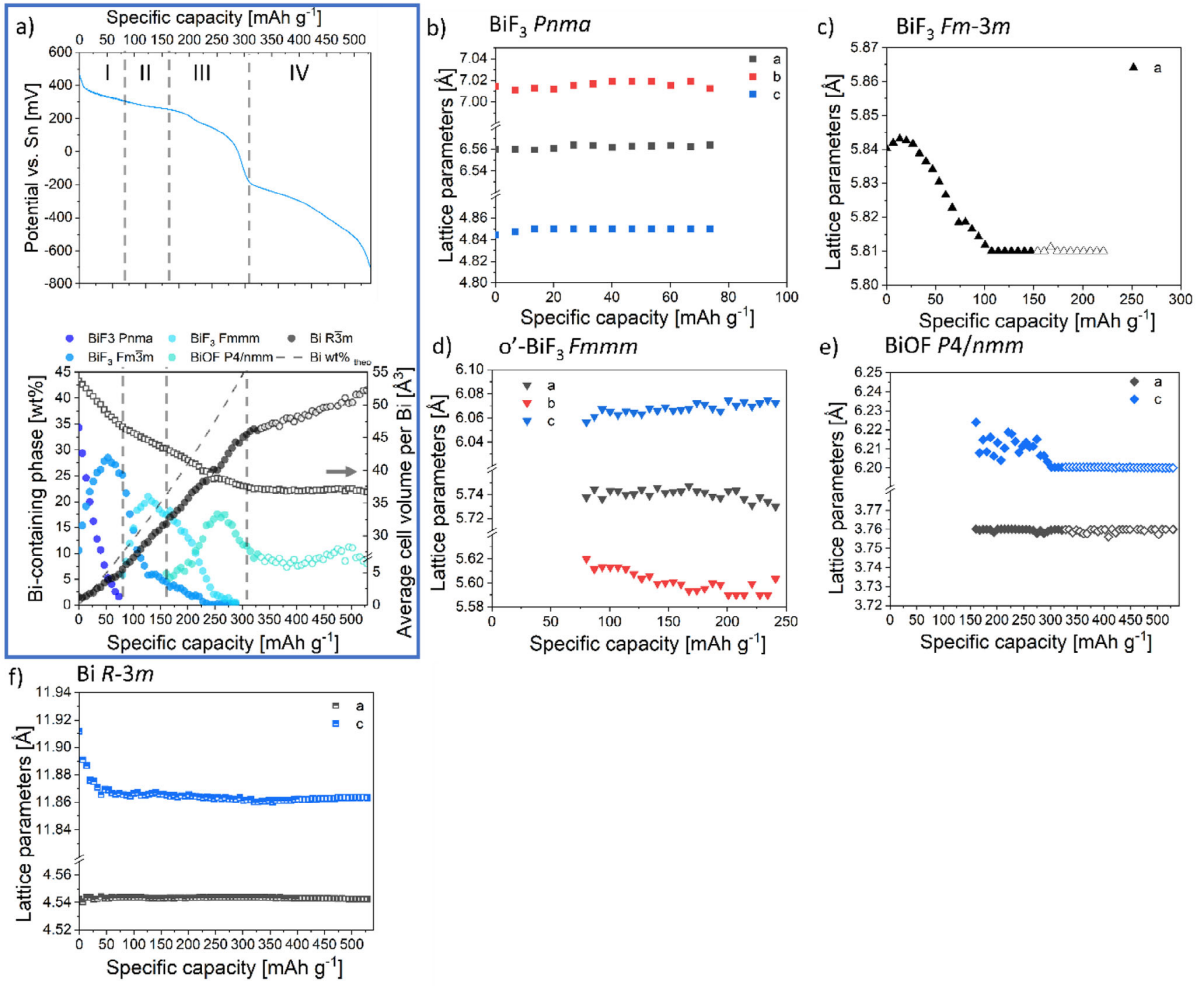
a) Cell BiF_3_|BaSnF_4_|Sn potential curve against specific capacity in Figure 4b and the corresponding weight fraction of Bi‐containing phases present in the BiF_3_ cathode composite and average unit cell volume per Bi ion during first defluorination at 100 °C, analyzed by batch Rietveld refinement on *operando* XRD results. Lattice parameters of the Bi‐containing phases extracted by Rietveld refinement against specific capacity. b) orthorhombic BiF_3_ (space group: Pnma), c) cubic BiF_3_ (space group: Fm‐3m), d) second orthorhombic o’‐BiF_3_ (fit in space group: Fmmm), e) tetragonal bismuth oxidefluoride BiOF (space group: P/4nmm), and f) rhombohedral metallic Bi (space group: *R‐*3*m*).

**Figure** **5**a–f shows the weight fraction of Bi‐containing phases, average unit cell volume per Bi ion as well as structure detail information of the Bi‐containing phases extracted as a function of specific capacity. Consistent with the qualitative descriptions of pattern changes given above, the progression of phase fractions aligns well with a continuous evolution for different intermediate phases. By correlating the weight fraction in Figure 5a and the potential curve, the defluorination process of BiF_3_ at 100 °C can be clearly understood as a series of four different stages, and the cell potential changes (plateau regions vs slope regions) can be understood with the following model:

At the beginning of defluorination, denoted by stage I, a rapid transition from orthorhombic BiF_3_ to cubic BiF_3_ is observed to be dominant; the fluoride defects seem to be mainly introduced into the cubic phase, and negligible compositional changes of the orthorhombic phase are indicated by the absence of changes of the lattice parameters, see Figure 5b. The increase of the amount of cubic phase agrees with the fact that defects represent a type of disorder and corresponding phase transitions are often observed at high temperatures, i.e., a structure in low symmetry always loses its precise distortion of coordinated polyhedra as soon as defects (vacancies) are being created and then transform into a higher symmetry supergroup of the original structure. In Figure 5c, a simultaneous decrease in lattice parameter *a* of c‐BiF_3_ is observed, indicating that the composition of c‐BiF_3_ is not constant. A complete disappearance of the o‐BiF_3_ phase (≈34.3 wt.% initially) accompanied by an increase of 17.9 wt.% (from 10.6 to 28.5 wt.%) of c‐BiF_3_ corresponds to 18% (54 mAh g^−1^) of the theoretical capacity of BiF_3_, whereas only 5 wt.% of Bi‐metal are detected in stage I. The deviation of the observed weight fraction of Bi‐metal and the expected value from the consumed orthorhombic BiF_3_ could be attributed to the poor crystallinity of Bi and some nanocrystalline which are not detectable by X‐ray at this stage. This is consistent with the initially considerable deviation of lattice parameter *c* of Bi to the value from the database (Figure 5c and *c* = 11.862(6) Å at room temperature from ICSD^[^ ^50^ ^]^), which comes from the nanocrystalline defect‐richer nature of the Bi‐metal at the beginning of the defluorination process. With defluorination the lattice parameter *c* value decreases gradually to the more ideal value known from literature at a capacity ≈60–70 mAh g^−1^, with the deviation most likely originating from thermal expansion. Overall, this observation in stage I can be simply explained in the sense that the phase transition with fast kinetics usually introduces an intermediate phase before a recrystallization takes place. As a consequence, the average unit cell volume per Bi atom in cathode composite reduces as shown in Figure 5a by a hollow square. We emphasize that the structural behavior of stage I was also well expressed in the ex situ study. A small difference is noted with respect to the phase fraction of o‐BiF_3_, which appears to exist also at higher states of discharge. This difference likely originates from the fact that c‐BiF_3_ is a high temperature modification and that the orthorhombic distortion likely re‐establishes on cooling from parts of the c‐BiF_3_.^[^ ^48^ ^]^

Upon defluorination, in Figure 5a, stage II is linked to the formation of o’‐BiF_3_ as the second intermediate phase. It is shown in Figure 5b, that a substantial decrease by 0.02 Å (0.34%) in lattice parameter *a* is observed for c‐BiF_3_ before symmetry lowering is triggered. As mentioned before, o’‐BiF_3_ is likely structurally related (face‐centered Bi‐substructure), only having slight compression on the *a* and *b* direction and extension along the *c* direction as compensation. During stage II, both c‐BiF_3_ and o’‐BiF_3_ change their lattice parameters, as shown in Figure 5c,d, most likely as the consequence of losing F^‐^ ions. The orthorhombic distortion increases, indicated by the continuous increase of the *c_Fmmm_*/*a_Fmmm_* ratio. A rather smaller volume change of the unit cell per Bi atom (3.7 Å^3^) is observed in stage II compared to that of stage I (6 Å^3^) (Figure 5a), even though the amount of Bi formed during this stage is approximately two times of that in stage I. This suggests that cycling within the capacity range of stage II is not a pure conversion mechanism and might also partly contain the intercalation‐type character. By exploiting this feature, one can potentially improve the cyclic stability of BiF_3_ cathode composite at such temperature conditions.

It has been discussed in our ex situ study that bismuth oxidefluoride, as the third intermediate phase, would be involved as soon as the reduced F/Bi composition from defluorination and the local enriched oxygen environment created by ions agglomeration through solid‐solid interphase diffusion matches certain values at which BiOF is thermodynamically more stable. In our *operando* XRD results the formation of BiOF referred to as stage III corresponds essentially to the ex situ results, both covering the specific capacity from ≈170 mAh g^−1^ to the theoretical capacity of BiF_3_ (302 mAh g^−1^). However, *operando* XRD addresses it with more detailed structure evolution information. As demonstrated in Figure 4, BiOF has a tetragonal unit cell, with Bi, O, and F ions generating ordered layer structures along the *c* direction. Figure 5e reveals that BiOF crystallizes initially with mostly the same lattice parameter *c* = 6.2240(2) Å and a slightly larger lattice parameter *a* = 3.7595(5) Å than the value (*a* = 3.746(9) Å, *c* = 6.226(1) Å)^[^ ^42^ ^]^ from database due to thermal expansion, for ongoing defluorination the lattice parameter *c* becomes smaller as a result of defluorination but *a* value stays similar. The significant deviation along the *c* direction during defluorination is due to the layer ordering of F^‐^ ions present in the structure which are being extracted upon defluorination. Nevertheless, stage III is mainly characterized by the disappearance of o’‐BiF_3_ and an increase amount of metallic Bi, which induces slightly larger volume changes per capacity change as observed in stage II, agreeing with a conversion mechanism.

Stage IV starts beyond the theoretical capacity of BiF_3_ (302 mAh g^−1^) until the measurement is stopped at 530 mAh g^−1^ (cell potential −700 mV), where the degradation of the electrolyte is observed to be dominant and contributes to the cell capacity. The evaluated structural information shows that ≈10 wt.% of BiOF remains from stage III and the stabilized lattice parameters for oxidefluoride and metallic bismuth in Figure 5e,f both indicate a stability of BiOF toward further reduction in stage IV. The apparent increases of phase fractions observed in Bi and bismuth oxidefluoride at the latter state of stage IV do not originate from the crystallization processes of Bi‐phases; their increase is coming from the fact that the phase fraction of the BaSnF_4_ phase decreases and the changes of the BaSnF_4_ show unfavorable overlap, i.e., misfit of reflection (102) of electrolyte at lower 2‐theta position (see refinement in Figure S8b, Supporting Information for scan No.80). One of the reasons for the observed unreactive oxidefluoride phase could be attributed to the strong Bi─O bond which requires way more negative cell potential to break and another one could be the loss of accessibility of oxidefluoride particles from other particles due to the volume change throughout the defluorination.

### 2.4 Charge Evolution of Bi and Sn Investigated by *Operando* XAS

In addition, *operando* XAS measurements were performed to separate ion exchange reactions (e.g., 2 F^‐^ for O^2‐^) which could lead to phase changes from redox‐mediated processes. Thus, these measurements allow the investigation of oxidation state changes in real time and can help address whether significant side reactions occur in the electrolyte. Here, we monitor the Bi‐L_III_ and Sn‐K edges during discharge at 100 °C and evaluate the oxidation state of Bi and Sn as well as the local environment around Bi up to a capacity of 300 mAh g^−1^ (Discharging curve in Figure S12a, Supporting Information). From the XANES spectra of the Sn K‐edge Figure S12b, Supporting Information), redox inactiveness of the electrolyte until 300 mAh ^‐^g^1^ (corresponding −380 mV cell potential; time constraints of measurement applied) is concluded as the spectra remain unaltered. Thus, changes of lattice parameters of the electrolyte phase in ex situ XRD must have a different origin, which we assume to originate from anion exchange reactions (exchange of O^2‐^ impurities for 2 F^‐^ between the different phases in the composite), according to

(1) $$\begin{array}{c} \mathrm{BaSn}F_{4-d}O_{d/2}+n\;\mathrm{Bi}F_{3-y}O_{y/2}\to \mathrm{BaSn}F_{4-d+z}O_{d/2-z}\\ +n\;\mathrm{Bi}F_{3-y-z/n}O_{y/2+z/n} \end{array}$$

**Figure 6:**
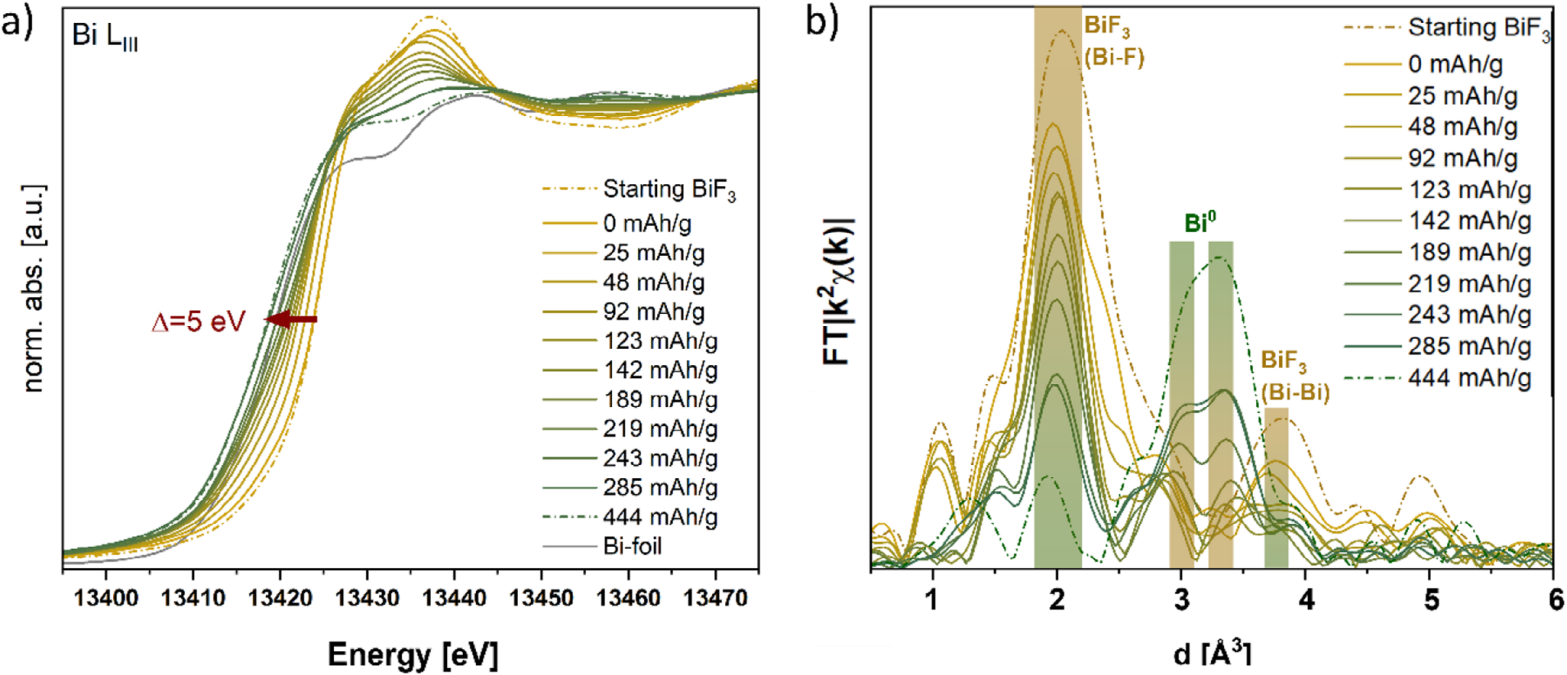
a) *Operando* XANES spectra of Bi K‐edge on recorded cell BiF_3_|BaSnF_4_|Sn while discharging operated by 20 uA cm^−2^ at 100 °C. b) Fourier transformed EXAFS spectra of Bi K‐edge. The reference spectrum of starting BiF_3_ refers to the ex situ measurement on the cathode side of an as‐prepared cell pellet at room temperature, and the reference spectrum for 444 mAh g^−1^ was also ex situ measured on another cell pellet which has been discharged in a Swagelok cell with the same heating condition and electrochemical operation.

In contrast, the XANES spectra recorded on the Bi K‐edge (**Figure** **6**a) show a continuous shift to lower energies (toward what is observed for an ex situ fully discharged cell pellet to a capacity of 444 mAh g^−1^ and Bi foil). From this a reduction of Bi from Bi^3+^ to Bi^0^ is concluded. The overall shift of ≈5 eV is in agreement with the edge energy difference of BiF_3_ (13 424 eV) and a Bi foil (13 419 eV). We emphasize that the spectra show clear shifts at the start of discharging, though only low amounts of metallic Bi can be detected in stage I (with large lattice parameter *c* as compared to literature references^[^ ^50^ ^]^). This can be attributed to the formation of nanocrystalline Bi in the early discharging stage, or the intermediate cubic BiF_3_ which contains certain amounts of lower valent Bi and defects.

For the analysis of the EXAFS region the Fourier transformed spectra were fitted with two different models, as shown in Figure S13 and Table S6 (Supporting Information) Model 1 reflects typical backscatterers obtained from a BiF_3_ crystal structure^[^ ^51^ ^]^ and consists of three F shells in distances of 2.20, 2.35 and 2.50 Å and three Bi shells at 3.89, 4.00 and 4.40 Å. Model 2 describes a mixture of BiF_3_ and metallic Bi. Beside the three F shells and one Bi shell (at 3.89 Å) from model 1 two Bi shells at distances of 3.0 and 3.5 Å were fitted, which are characteristic distances in metallic Bi.^[^ ^50^ ^]^ Besides for the fit of the BiF_3_ reference model 1 exhibited better fitting errors for the discharge capacity up to 123 mAh g^−1^ (Figure S14, Supporting Information). During this time the number of F backscatterers decreases continuously from 8.1 to 4.3 following the reduction to Bi^0^ visible in XANES. Starting with the spectrum measured at a capacity of 142 mAh g^−1^) the application of model 2 resulted in lower error values (Figure S14, Supporting Information) and the number of F backscatterers continues decreasing to 2.5 with a capacity of 285 mAh g^−1^ (end of *operando* measurements) and to 1.3 for the ex situ measured discharged sample (to 444 mAh g^−1^). In the same time the contributions of metallic Bi increase as can be seen in Figure 6b, reflecting the formation of Bi^0^.

During the discharging, the number of F backscatterers fluctuates in each shell and the obtained Debye‐Waller factor is comparably high, which indicates significant disorder and complex structural changes. This likely originates from short Bi‐X distances within o‐BiF_3_, c‐BiF_3_, o’‐BiF_3,_ and BiOF; due to the complexity of the phases formed, a more detailed deconvolution is not possible.

## 3 Conclusion

In this study, we successfully designed an *operando* cell for a laboratory X‐ray diffractometer to monitor the real‐time structural evolution of BiF_3_ cathode materials during the first discharge process at elevated temperatures. On the contrary to a straight reconstructive transformation model between the most stable modification of BiF_3_ (*Pnma*) and Bi (*R*‐3*m*) our findings reveal that BiF_3_ undergoes a multi‐step phase transformation from o‐BiF_3_ to c‐BiF_3_ and then o’‐BiF_3_ before the formation of Bi metal, showing an intercalation‐type defluorination mechanism. The observation of Bismuth oxidefluoride formation at a later defluorination state, attributed to the oxygen impurity from the solid electrolyte, highlights the influence of the synthesis method on cell performance.

*Operando* XAS measurements confirmed the continuous reduction of the Bi oxidation state and the emergence of different intermediate phases during the first defluorination and the redox inactiveness of the electrolyte BaSnF_4_ up to 300 mAh g^−1^ (−380 mV against Sn/SnF_2_). Rietveld analyses of *operando* XRD results provided the dependence of phase fractions as well as crystal structure information on the cell potential (specific capacity), enabling the development of a cell potential changes model for the first defluorination of BiF_3_.

Additionally, our study demonstrates the significant role of the solid electrolyte BaSnF_4_ in the transport process of both fluoride ions and oxygen impurities within the BiF_3_ cathode composite. The wide potential window of BaSnF_4_ and its degradation behavior at potentials below −200 mV against the Sn/SnF_2_ anode were also elucidated, implying the potential application of BaSnF_4_ with high‐voltage electrode materials.

Overall, *operando* XAS and *operando* XRD provide detailed insights into the phase transformations occurring during the first discharge process of BiF_3_ electrode materials. The observed influence of the impurity transport process is crucial for understanding the capacity fading phenomena and for the further development of high‐performance fluoride ion batteries. Future work should focus on: i) improving the purity of solid electrolytes and exploring alternative synthesis methods to enhance the stability and efficiency of these energy storage systems, ii) minimizing the contact loss among particles, and reducing the density of solid‐solid interfaces by applying stacking pressure during cell operation to suppress BiOF formation and improve the cycling performance.^[^ ^52^ ^]^

## 4 Experimental Section

### Synthesis

Barium fluoride (BaF_2_) (99.99%) and tin fluoride (SnF_2_) (99%) (XRD patterns in Figure S1, Supporting Information) from Sigma–Aldrich were used for synthesizing electrolyte BaSnF_4_ by mechanical milling and post soft annealing. All starting materials were dried in a vacuum furnace inside an argon‐filled glovebox to properly remove any absorbed moisture. Stoichiometric amounts (≈3 g in total) were sealed in a ZrO_2_ milling vial (50 mL) with ZrO_2_ milling balls (5 mm diameter, ball‐to‐powder ratio of 17:1) under argon atmosphere in the glovebox and milled at 600 rpm for 4 h. After ball milling, the powder mixture was annealed at 300 °C for 2 h under dynamic vacuum (10^−2^ mbar) using a *Büchi* Glass Oven B‐585. The mechanical milling and the annealing process were repeated three times to enhance the doping process. Bismuth trifluoride (BiF_3_) (99%), Sn nanopowder (>99%, <150 nm particle size (SEM)), and carbon nanofibers (CNF) (>98%) from Sigma–Aldrich were used to prepare the BiF_3_ cathode and Sn anode composites, as described by Reddy et al.^[^ ^18^ ^]^ The cathode composite consisted of 40 wt.% BiF_3_, 50 wt.% BaSnF_4_, and 10 wt.% CNF, while the anode composite contained 50 wt.% Sn, 40 wt.% BaSnF_4_, and 10 wt.% CNF. Again, BiF_3_ and CNF were dried at 190 °C under dynamic vacuum for 24 h using the vacuum furnace before the synthesis process.

### Electrochemical Measurements

**Figure 7:**
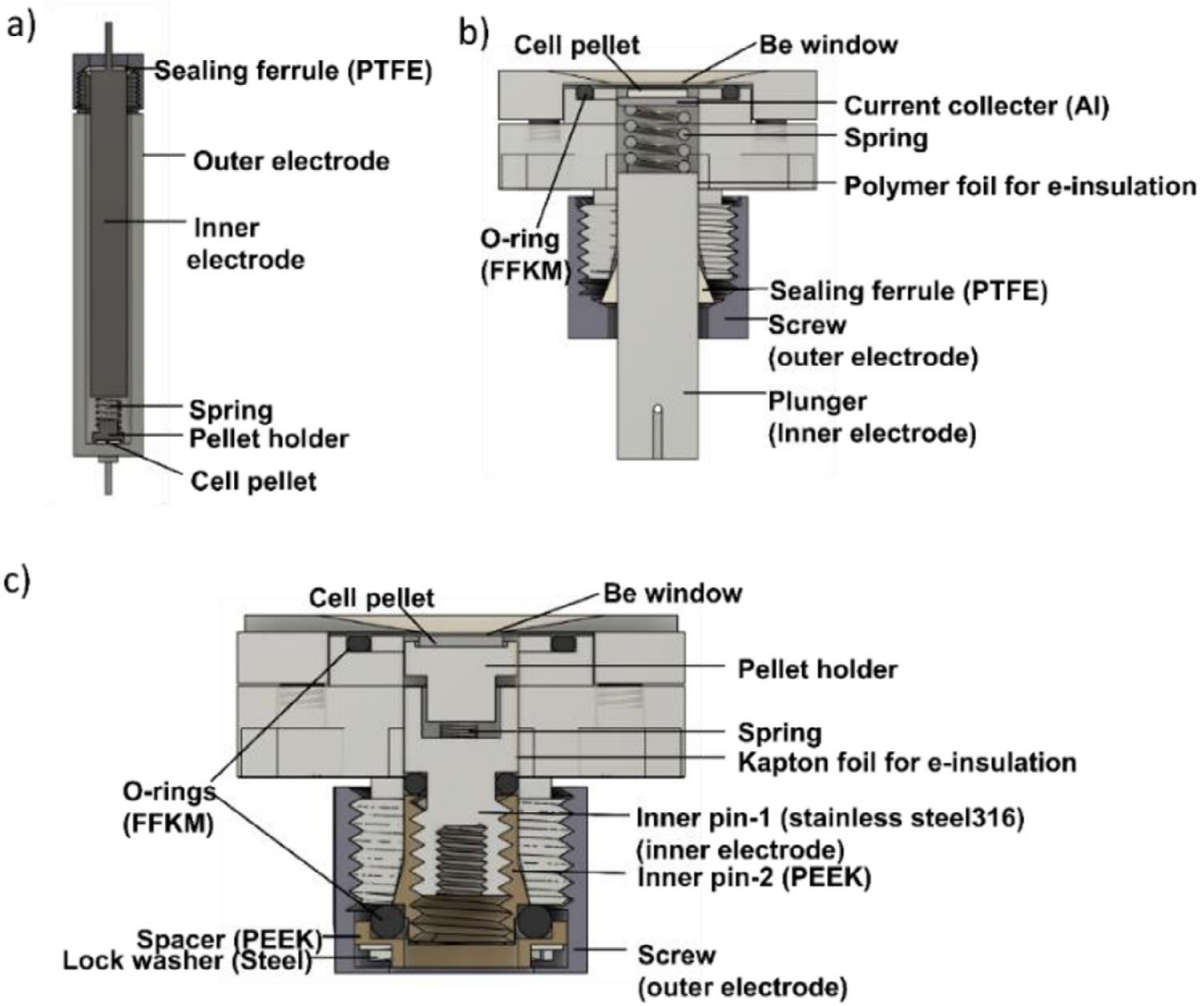
Cell configuration and schematic sketches of a) the Swagelok‐type cell for ex situ investigation, the modified Swagelok‐type cell designs with Be window facing up in two sizes for b) operando XAS measurement and for c) operando XRD operation.

Cell pellets were prepared in a three‐layer cell configuration by uniaxial pressing anode composite, electrolyte, and cathode composite using a *Specac* Atlas 25T manual hydraulic press in an argon‐filled glovebox (2 t for 90 s). The mass loadings of electrolyte, anode composite, and cathode composite were 200, 10, and 5 mg, respectively, i.e., the anode composite was used in excess as compared to the cathode composite. The galvanostatic cycling was performed at 100 °C with a current density of 20 µA cm^−2^ (unless specified otherwise) on potentiostats from *Biologic Science Instruments* (VSP or VMP‐300 for cycling only and SP‐150, VSP‐300 for *operando* XAS and XRD measurements, respectively). The specific capacities were calculated based on the weight of active material in the cathode composite (BiF_3_). Due to the different electrochemical operations and measurement limitations (space of set up, time, etc.) for ex situ and *operando* characterization techniques, three types of cell designs were custom‐made and used. **Figure** **7**a shows a typical high‐temperature Swagelok type cell,^[^ ^18^ ^]^ which is used for charging/discharging cell pellets for ex situ analysis. For *operando* XRD/XAS measurements during galvanostatic cycling operation, two Swagelok‐type cells of compact design were used due to the different space limitations of measurement devices. As shown in Figure 7b,c,^[^ ^28^ ^]^ those cells possess a Be window on one side acting as a current collector and behaving stable at elevated temperatures (100 °C in this study). The sealing integrity of the cells was confirmed by the stable ionic conductivity of solid electrolyte at cell operating temperature, given its observed sensitivity to moisture (see Figure S16, Supporting Information and accompanying comments).

### X‐Ray Diffraction and Rietveld Analysis

The synthesized electrolyte, cathode, and anode composites as well as cell pellets after galvanostatic cycling were characterized by X‐ray diffraction (XRD) in Bragg‐Brentano geometry). In this study, a *Rigaku SmartLab* diffractometer with Cu *Kα_1,2_* radiation (40 kV, 30–50 mA) and a HyPix‐3000 detector was used.

For ex situ X‐ray diffraction experiments, powder samples or cell pellets at different states of charge were placed in low background airtight sample holders inside an argon‐filled glovebox. XRD patterns were recorded at room temperature using an incident slit size of 0.3° in the 2‐theta range from 10° to 80°, with a total measurement time of 2 h 35 min and a step size of 0.005°.

*Operando* XRD patterns were recorded at 100 °C on the cathode side of pellets while galvanostatic discharging being performed. The 2‐theta range was limited to 21.5°–40.5° with a scan time of 26 min as a compromise to record a representative and suitable angular range for later data analysis while optimizing the measurement time for both time resolution and signal‐to‐noise ratio. An incident slit size of 0.6° and a step size 0.005° were used in order to produce good signal intensities and data resolution. To obtain sufficiently time‐resolved data allowing for both phase analysis and quantification, a loop measurement was programmed to record XRD patterns during the galvanostatic discharge process, at 1‐h intervals. Therefore, the corresponding specific capacity difference between the two patterns obtained in this work is ≈7 mAh g^−1^. An image of the measurement setup is shown in Figure S6a (Supporting Information). Before experiments started, the z position (height) of the *operando* XRD cell was aligned at room temperature, following a room temperature measurement in order to check the initial composition and structure. After the cell temperature stabilized at 100 °C for 1 h, the discharge process as well as the loop measurement of XRD patterns were initiated.

Rietveld analysis of the diffraction data was performed using TOPAS V6^[^ ^53^ ^]^ using a fundamental parameters approach as described in literature,^[^ ^44^ ^]^ with the instrumental broadening being derived from a reference scan on a NIST standard of LaB_6_ (660a). The pattern corresponding to the solid electrolyte phase is difficult to refine (asymmetric peak shapes originating from the inhomogeneous distribution of Ba/Sn within the particles, requiring multiple similar phases to refine the reflection shapes appropriately; for more information see paragraphs 1–3 in chapter 0) and was learned from a scan of the pure electrolyte to minimize refinable parameters for the analysis of the complex phase mixtures on initiating the reduction of BiF_3_. Thus, only one parameter was allowed for the scaling of the electrolyte phases and marginal reflection shifts due to thermal expansion were allowed for the in situ analysis by applying appropriate constraints on the scaling and lattice parameters of the individual electrolyte phases. Structural models, as reported in the literature,^[^ ^40^, ^42^, ^50^, ^54^ ^]^ were used for the different modifications of BiF_3_, Bi, and BiOF without adjusting the atomic positions within the refinements, but allowing for the refinement of lattice parameters. The structural model for the new orthorhombic modification o’‐BiF_3‐x_ was derived as described in the discussion on *operando* results. Two Voigt functions were used to account for angular‐dependent broadening effects due to crystallite size and microstrain in the individual phases; reasonable parameter limits derived from patterns with maximum phase quantities were used in order to stabilize the refinements with reduced phase fractions of the individual phases. The thermal displacement parameters of all atoms of all phases were constrained to be identical to minimize quantification errors.

In this work, batch Rietveld analyses were performed on *operando* XRD patterns to gain a deeper understanding of the structural correlations underlying the reaction mechanism and the relationship between the transition process, Bi formation kinetics, and the cell potential changes. As marked in Figure S8 (Supporting Information), four patterns from scan No. 1, 13, 30, and 45 (text in red color) were selected to be manually refined and the results of them were used as the boundary values for four groups of patterns which are categorized by phase transition stages observed. The batch analysis was proceeded by Python codes which apply the pre‐refined boundary values in *Topas* V6 software to the corresponding group of patterns and repeat the fitting process with constraints carefully set on parameters. The reliability of the results was judged not only by a good R_wp_ value of each refinement but also by comparing them to manually refined values of other patterns randomly selected from the data set (see Figure S8, Supporting Information). More details such as R_w_ values of batch analysis are provided in Figure S9 (Supporting Information).

### X‐Ray Absorption Spectroscopy (XAS)

*Operando* XAS experiments at the Bi L_III_‐edge (13 419 eV) and the Sn K‐edge (29 200 eV) on a cell pellet of BiF_3_|BaSnF_4_|Sn at 100 °C during galvanostatic discharging were carried out at the DESY (Deutsches Elektronen‐Synchrotron) beamline P65 in Hamburg, Germany. The current density used for galvanostatic discharging is 20 µA cm^−2^. An image of the measurement setup is shown in Figure S6b (Supporting Information). The measurements were performed using a double‐crystal monochromator — Si(111) for Bi, Si(311) for Sn measurements — with a maximum synchrotron beam current of 100 mA in continuous mode (300 s per spectrum). All spectra were recorded in fluorescence mode due to the operando setup. Additionally, for measurements at the Bi edge a Co filter (3 µm thickness) was applied to reduce interference radiation on the detector. For energy calibration, a Bi foil and a Sn foil were measured in advance and after sample spectra to account for possible energy shift due to monochromator movements. The edge energies were determined by the first maximum in the spectrum derivatives. For comparison and analysis of the overall energy shift range during galvanostatic discharging, ex situ XAS spectra of another fresh‐prepared cell pellet (denoted as starting BiF_3_ in Figure 6a,b) and a cell sample discharged to 444 mAh g^−1^ (also investigated by ex situ XRD) were recorded in advance.

The fresh‐prepared cell pellets for *operando* and ex situ measurements were vacuum sealed during transport to the beamline and the complete handling of the samples (cell assembling, cell pellet sealing) was carried out under an inert atmosphere. During the measurement of the ex situ samples the pellets were sealed in Kapton tape to minimize the air‐exposure.

## Conflict of Interest

The authors declare no conflict of interest.

## Author Contributions

H.C. developed the operando XRD cell setup, executed the synthesis of the precursors, preparation of the electrochemical cells and samples for further characterization measurements such as elemental analysis, XRD and XAS measurements, performed ex situ and operando XRD measurements, and electrochemical part of operando XAS measurement, analyzed of XRD and GCPL data including plotting of data, wrote the manuscript. R. S. performed X‐ray absorption spectroscopy and analyzed XAS data including plotting of data with guidance from M. B. J. C. provided Operando XAS cell for measurement. Y. T. performed an elemental analysis with guidance from R. N. K. W. contributed to operando XRD measurement such as introducing installation of measurement setup in laboratory X‐ray diffractometer and discussing diffraction measurement strategy. O.C. guided and supervised the project, and contributed to writing the manuscript.

## Supporting information



Supporting Information

## Data Availability

The data that support the findings of this study are available in the supplementary material of this article.
