# Supplementary material for: Revealing an Intercalation Nature of High‐Capacity Conversion Cathode Materials for Fluoride‐Ion Batteries by Operando Studies
Source: Small Methods. 2025 Jun 4;9(8):2500374. doi: 10.1002/smtd.202500374 (PMC12391626; doi:10.1002/smtd.202500374)
Supplement: Supplementary file 1 — Supporting Information [file SMTD-9-2500374-s001.pdf]

small methods

Supporting Information

for *Small Methods*, DOI 10.1002/smtd.202500374

Revealing an Intercalation Nature of High-Capacity Conversion Cathode Materials for Fluoride-Ion Batteries by Operando Studies

*Hong Chen, Roland Schoch, Jean-Noel Chotard, Yannick M. Thiebes, Kerstin Wissel, Rainer Niewa, Matthias Bauer and Oliver Clemens**

Supplementary Information for

Revealing an Intercalation Nature of High-Capacity Conversion Cathode Materials for Fluoride-Ion Batteries by Operando Studies

Hong Chen[a], Roland Schoch[b], Jean-Noel Chotard[c], Yannick M. Thiebes[d], Kerstin Wissel[a], Rainer Niewa[d], Matthias Bauer[b], Oliver Clemens[a]*

[a] University of Stuttgart, Institute for Materials Science, Materials Synthesis Group, Heisenbergstraße 3, 70569 Stuttgart, Germany

[b] Paderborn University, Institute for Inorganic Chemistry and Center for Sustainable Systems Design (CSSD), Warburger Str. 100, 33098 Paderborn, Germany

[c] Université de Picardie Jules Verne, Laboratoire de Réactivité et de Chimie des Solides, CNRS-UMR 7314, 80039 Amiens, France

[d] University of Stuttgart, Institute for Inorganic Chemistry, Pfaffenwaldring 55, 70569 Stuttgart, Germany

* Corresponding Author:

Prof. Dr. Oliver Clemens

Email: oliver.clemens@imw.uni-stuttgart.de

Fax: +49 711 685 51933

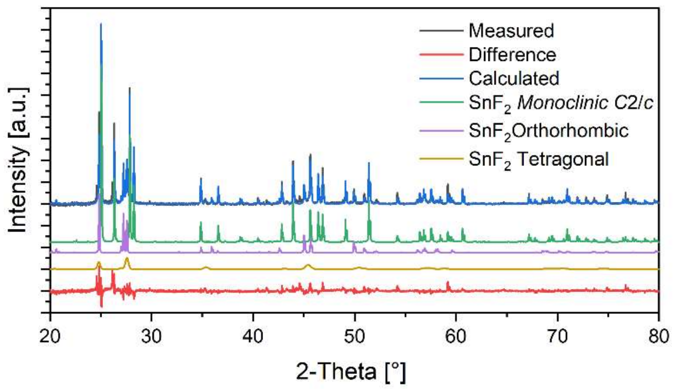

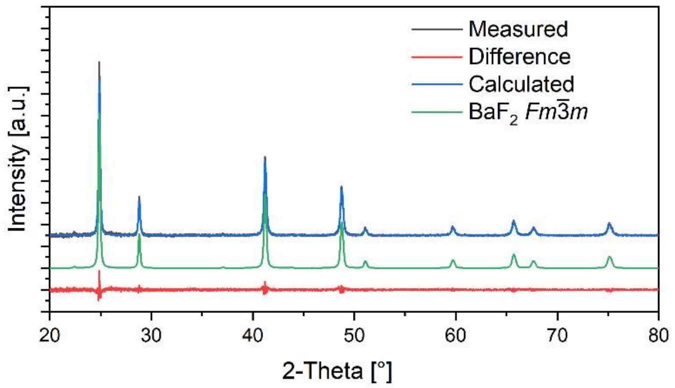

Figure S 1 XRD pattern of dried precursor for synthesis of $BaSnF_4$. top SnF_2, bottom BaF_2

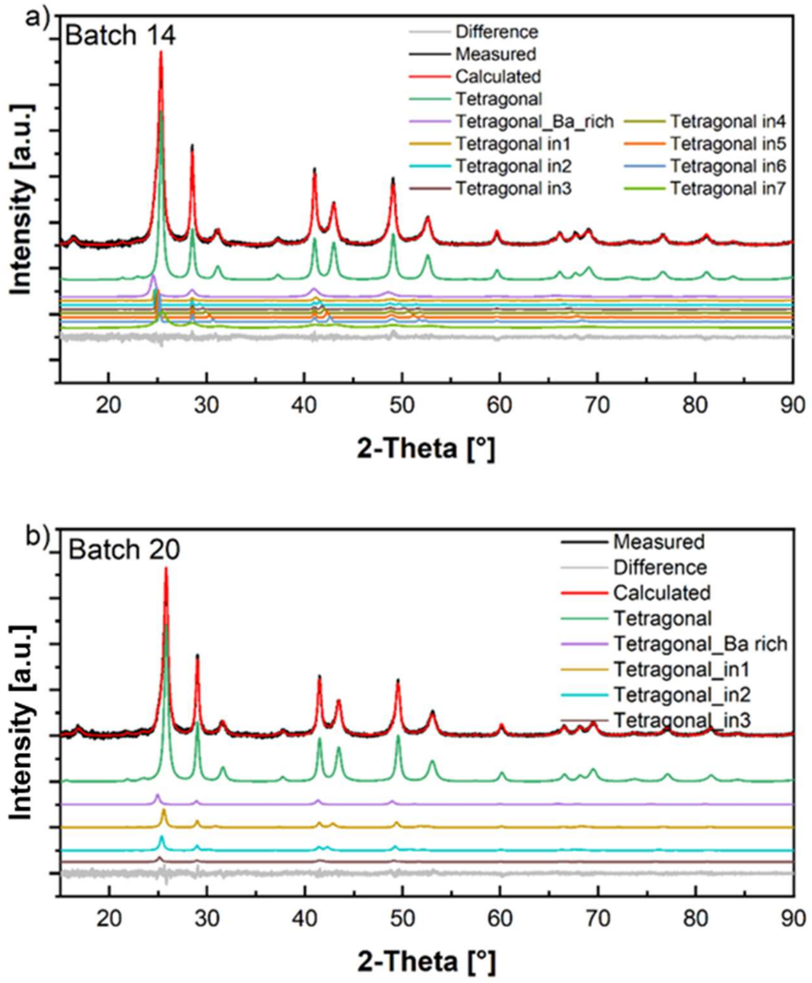

Figure S 2 XRD pattern of tetragonal $BaSnF_4$ batch 14 (top)and batch 20 (bottom) prepared by three times ball milling and post soft annealing process analyzed by Rietveld refinement method.

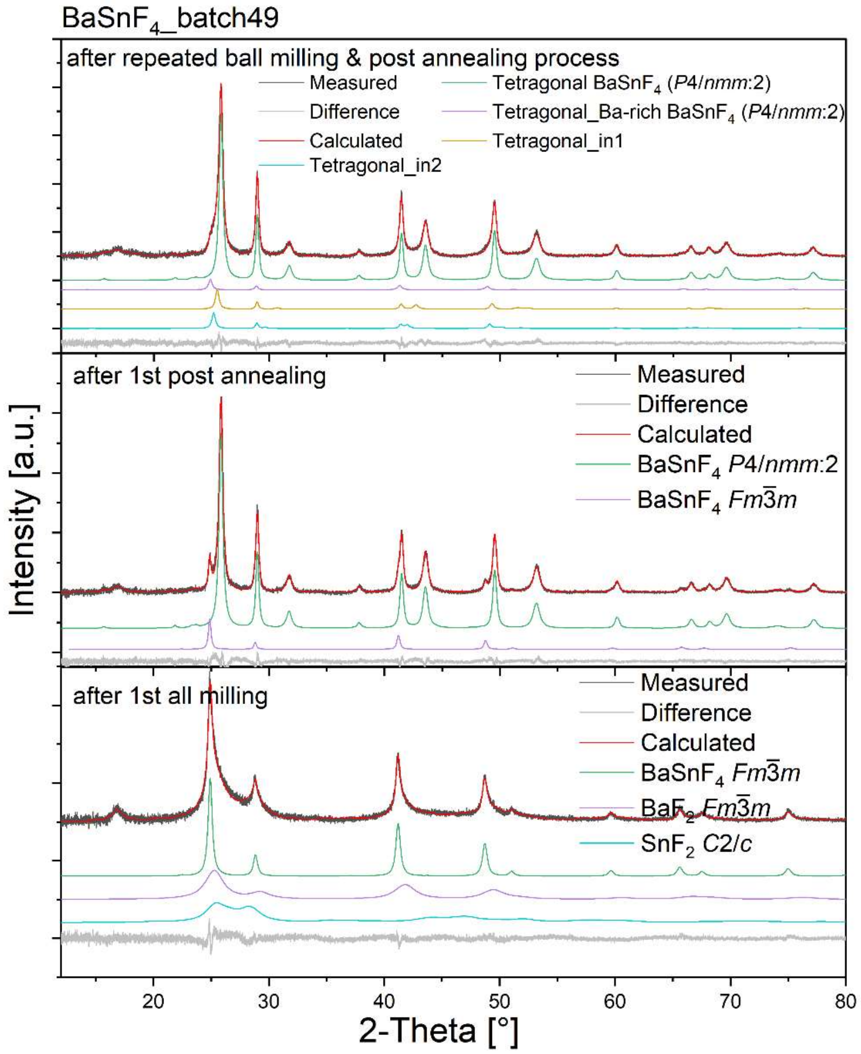

Figure S 3 Improving composition homogeneity in tetragonal $BaSnF_4$ by repeating the ballmill&post-annealing process. Bottom: cubic $BaSnF_4$ and unreacted BaF_2 observed in powder after 1st ball milling. Middle: powder after 1st annealing process has co-present tetragonal $BaSnF_4$ and cubic $BaSnF_4$. Top: $BaSnF_4$ after repeated ball-milling and post-annealing being performed.

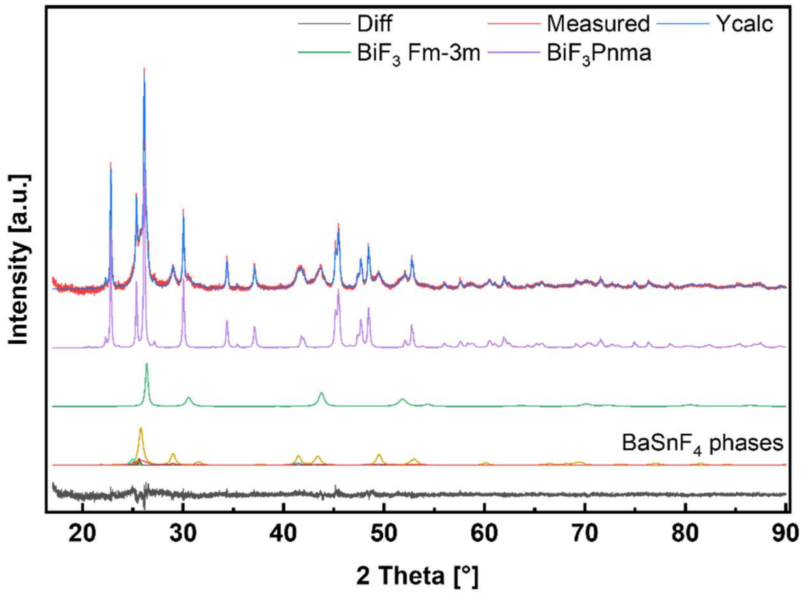

Figure S 4 XRD pattern of as-prepared BiF_3 cathode composite (with $BaSnF_4$ of batch14) analyzed by Rietveld refinement method.

Table S 1 Weight fraction and lattice parameters of BiF_3 modifications in dried pristine and as-prepared cathode composite (CC) by Rietveld analysis.

**Modifications of BiF_3**	**Phase fraction in dried pristine**	**Phase fraction in CC**	**Lattice parameters**
Fm-3m	14.75 wt %	8.11 wt %	In dried pristine: a (Å) = 5.8501(3)
			In composite: a (Å) = 5.8463(3)
Pnma	82.25 wt %	42.24 wt %	In dried pristine: a (Å) =6.56219(4); b (Å) =7.01575(4); c (Å) =4.83966(3)
			In composite: a (Å) =6.56348(14); b (Å) =7.01868(18); c (Å) =4.84102(14)

Table S 2 Elemental analysis on cathode active material BiF_3 and individual other ingredients (t-$BaSnF_4$ and CNF as well as pristine materials for electrolyte preparation).

**Sample name**	**average O content (wt%)**	**error (wt%)**
BiF_3 cathode composite_prepared with $BaSnF_4$ batch27	1.4	0.173
BaF_2 dried at 190 °C 20 h	0.394	0.0147
SnF_2 dried at 120 °C 20 h	0.399	0.00697
BiF_3 dried at 190 °C 20 h	0.776	0.368
$BaSnF_4$ batch14	0.916	0.0921
$BaSnF_4$ batch27	1.08	0.0367
Carbon nano fiber dried at 190 °C 20 h	0.291	0.0201

Table S 3 Partially transition from o-BiF_3 to c-BiF_3 observed in operando measurement.

			Values from database (RT)	As-prepared BiF_3 composite (from $BaSnF_4$ batch46, RT)	In-situ XRD cell (BiF_3 cathode side) after alignment (RT)	In-situ XRD cell (BiF_3 cathode side) heating at 100°C for 1 h
t-$BaSnF_4$	a (Å)		4.345(1)	4.3533(4)	4.360(2)	4.360(2)

P4/nmm	c (Å)	ICSD 166207	11.228(1)	11.294(2)	11.310(12)	11.31(1)
Weight fraction (wt%) of electrolyte in the composite				47.78	42.79	44.27
BiF_3 *Fm-3m*	a (Å)	ICSD 24522	5.861	5.8510(3)	5.8382(7)	5.8408(5)
weight fraction (wt%) of c-BiF_3 in the composite				10.01	6.41	10.74
Calculated weight fraction of c-BiF_3 in Bi-containing phases				19.17	13.22	23.18
BiF_3 *Pnma*	a (Å)	ICSD 1269	6.5614(4)	6.56333(13)	6.5620(5)	6.5597 (6)
	b(Å)		7.0153(5)	7.01802(14)	7.0163(6)	7.0145(8)
	c (Å)		4.8414(3)	4.84196(11)	4.8407(3)	4.8445(3)
weight fraction (wt%) of o-BiF_3 in the composite				42.21	42.07	35.59
Calculated weight fraction of o-BiF_3 in Bi-containing phases				80.83	86.78	76.82

Table S 4 Temperature stability of BiF_3 cathode composite (prepared with $BaSnF_4$ batch20). Heating on cathode composite powder was performed under argon inside an argon-filled glovebox at 100 °C for 50 h and 75 h.

			Values from database (RT)	as-prepared BiF_3 cathode composite (with $BaSnF_4$ batch20, RT)	BiF_3 cathode composite after heating at 100 °C for 50 h (Argon)	BiF_3 cathode composite after heating at 100 °C for 75 h (Argon)
t-$BaSnF_4$	a (Å)	ICSD 166207	4.345(1)	4.3506(2)	4.3515(3)	4.3507(3)
P4/nmm	c (Å)		11.228(1)	11.3100(3)	11.3100(18)	11.3100(17)
Weight fraction (wt%) of electrolyte in the composite				53.31	55.29	54.99
BiF_3 *Fm-3m*	a (Å)	ICSD 24522	5.861	5.8446(4)	5.8445(3)	5.8458(3)
weight fraction (wt%) of c-BiF_3 in the composite				8.54	9.43	7.95
Calculated weight fraction of c-BiF_3 in Bi-containing phases				18.72	21.09	17.65
BiF_3 *Pnma*	a (Å)	ICSD 1269	6.5614(4)	6.56378(9)	6.56548(10)	6.56453(9)
	b(Å)		7.0153(5)	7.01894(11)	7.01655(14)	7.01776(11)
	c (Å)		4.8414(3)	4.84317(5)	4.84310(6)	4.84212(5)
weight fraction (wt%) of o-BiF_3 in the composite				37.07	35.28	37.07
Calculated weight fraction of o-BiF_3 in Bi-containing phases				81.28	78.91	82.34

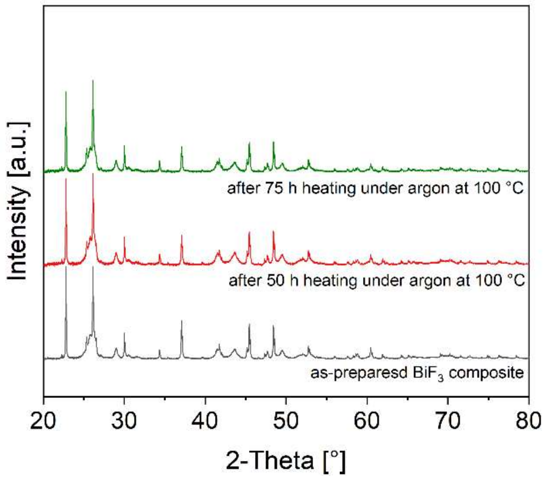

Figure S 5 Comparison of XRD patterns of BiF_3 cathode composite (prepared with $BaSnF_4$ batch20) after heating test. Heating on cathode composite powder was performed under argon inside an argon-filled glovebox at 100 °C for 50 h and 75 h.

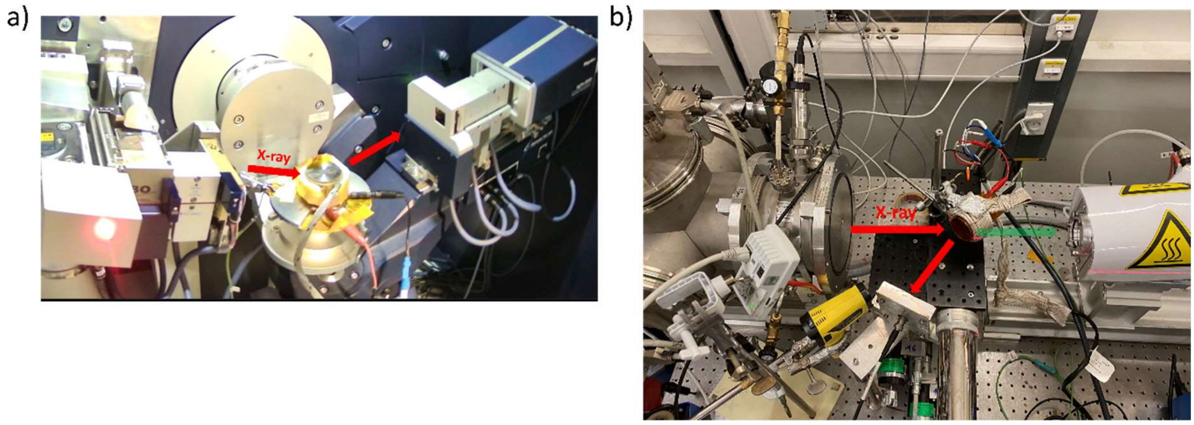

Figure S 6 experimental setup for Operando experiments. a) Operando XRD measurement (performed in reflection geometry) with Rigaku Smart Lab laboratory X-ray diffractometer; b) Operando XAS measurement (performed in fluorescence geometry) in DESY.The heating was conducted using a heating ring (for operando XRD) or a heating band (for operando XAS) with a thermocouple fixed next to the cell by Kapton foil and Kapton tape (also serving as electrical insulation)., The set temperature was calibrated by performing EIS measurement on electrolyte $BaSnF_4$, and comparing the total resistance value to the reference value obtained in Bio-Logic MTZ-35 and ITS-e at 100 °C.

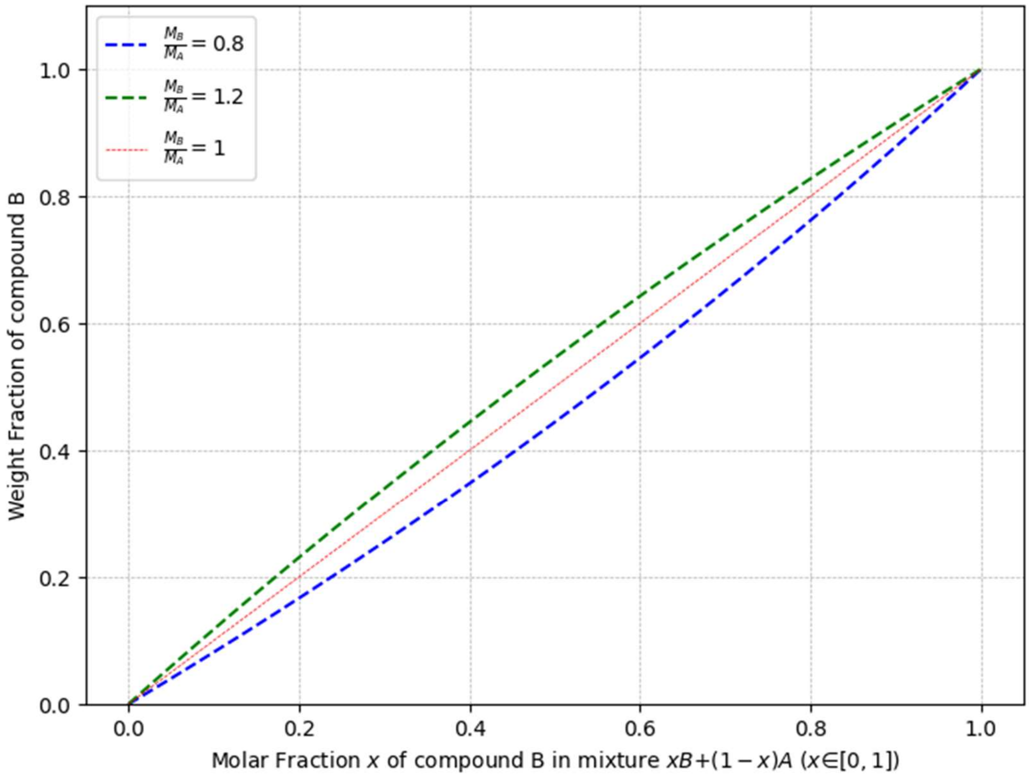

Figure S 7 Calculated weight fraction of compound B increases as molar fraction increases in a mixture of xB+(1-x)A 0≤x≤1, for different molecular mass ratio of compound B to compound A(plot by python).

a)

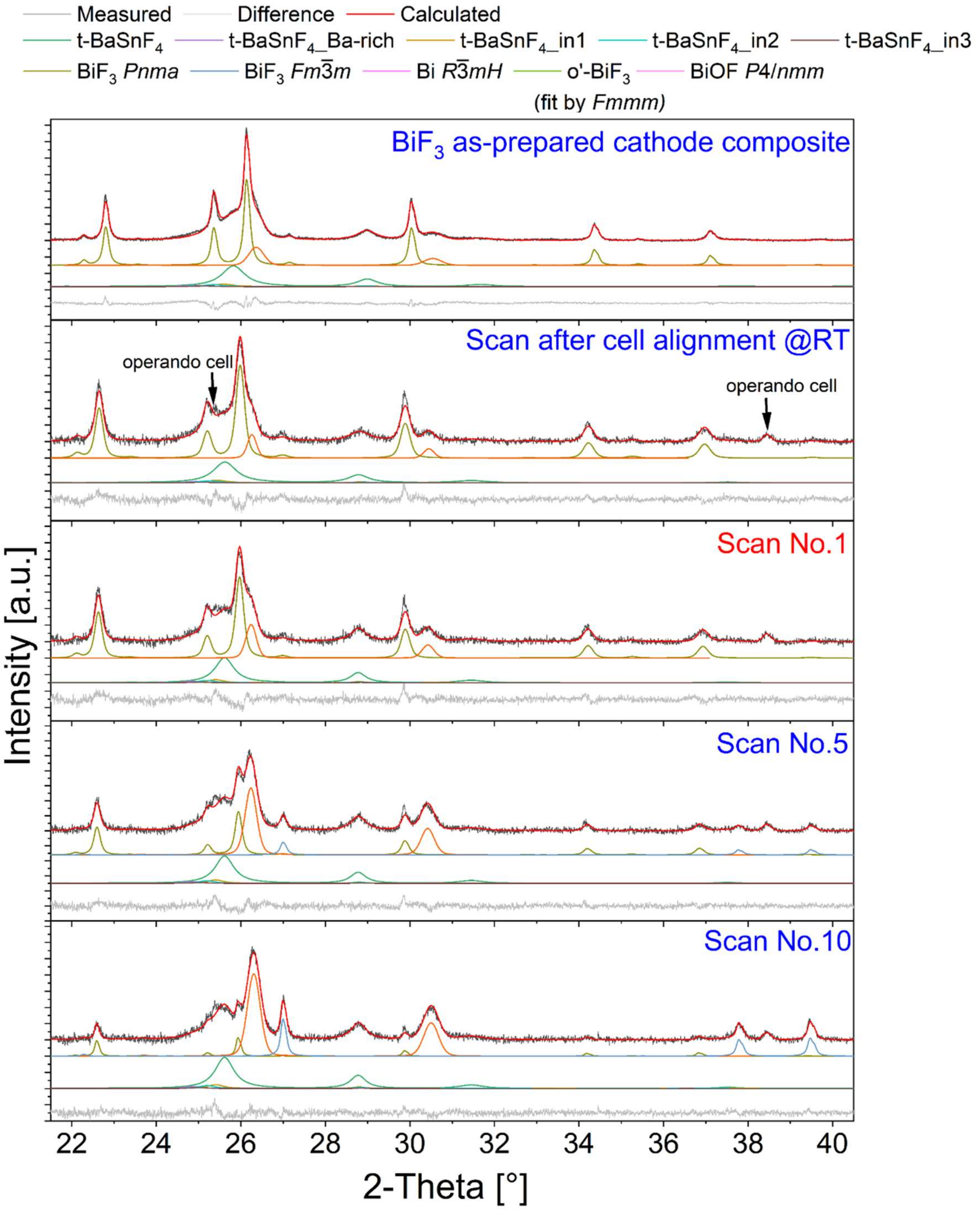

Measured
Difference
Calculated
t-BaSnF4
t-BaSnF4_Ba-rich
t-BaSnF4_in1
t-BaSnF4_in2
t-BaSnF4_in3
BiF3 Pnma
BiF3 Fm3̄m
Bi R3̄mH
o'-BiF3 (fit by Fmmm)
BiOF P4/nmm
BiF3 as-prepared cathode composite
Scan after cell alignment @RT
operando cell
operando cell
Scan No.1
Scan No.5
Scan No.10
Intensity [a.u.]
2-Theta [°]
22
24
26
28
30
32
34
36
38
40

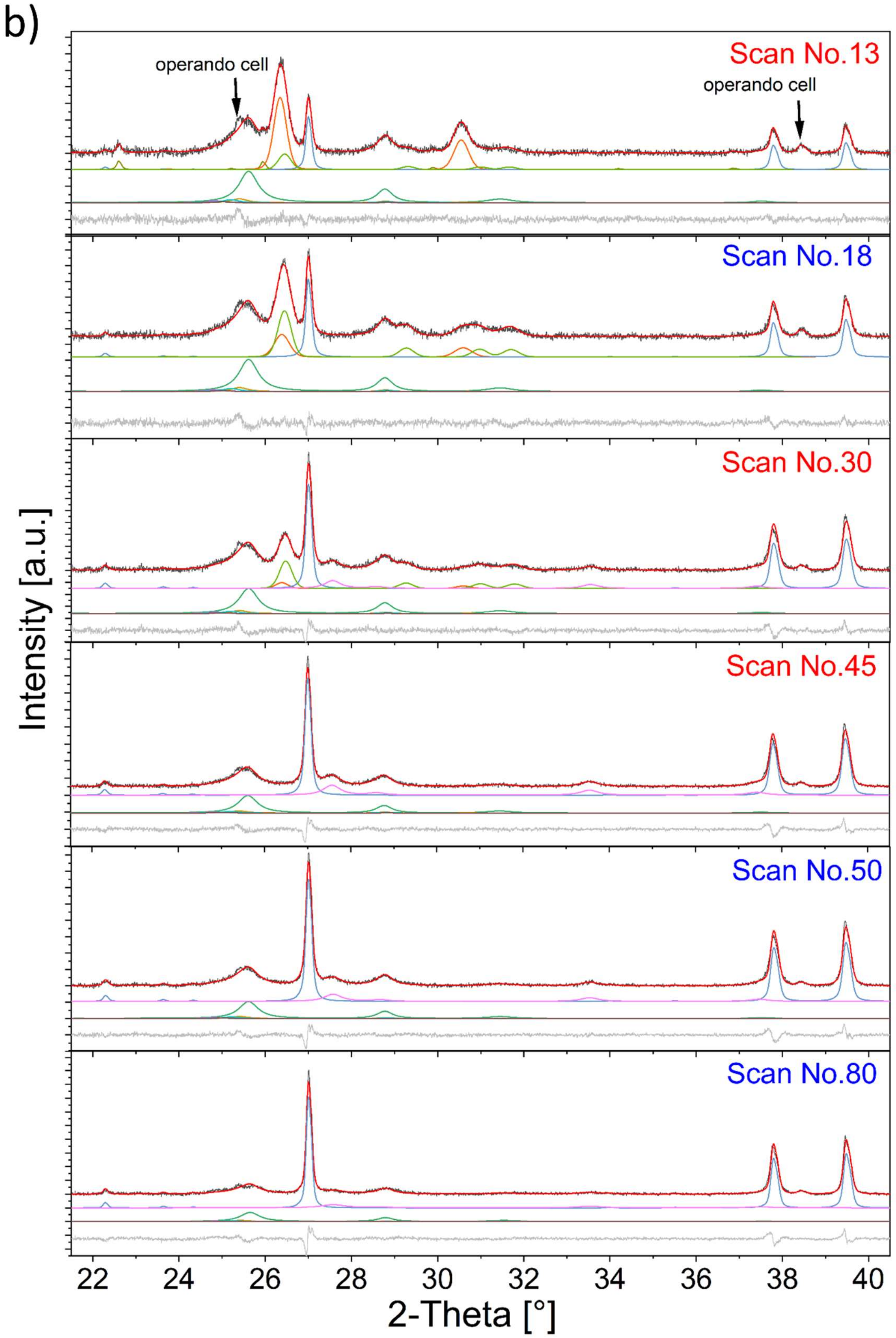

Figure S 8 Selected XRD patterns fit by Rietveld analysis (manually fitting). Fitting result of scan data numbered in red color are used as boundary values for four groups of batch refinement. signals marked by black arrows come from operando cell setup (BeO (100) at 2-theta=38.5°). a) XRD pattern and fitting result of as-prepared BiF_3 cathode composite (powder) measured at scan rate 1°/min and different scans of cell on cathode side during operando XRD measurement and their fitting a) cell before heating and scan No. 1, 5, 10 since discharging starts, with scan rate 1°/min). b) continue from a) scan No. 13, 18, 30, 45, 50, 80, with scan rate 1°/min.

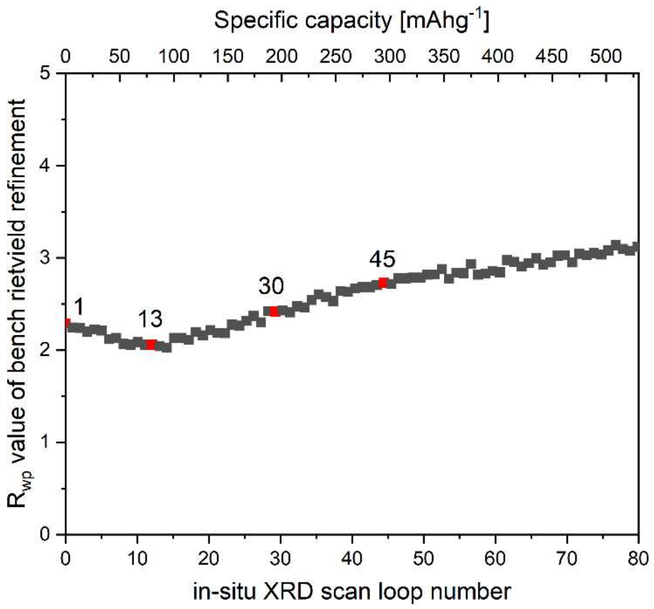

Figure S 9 R_{wp} values for bench Rietveld refinement. Scan number 1,13,30,45 were selected and manually refined as initial values for refining corresponding group of patterns, for which the same constraint conditions for refinement were applied.

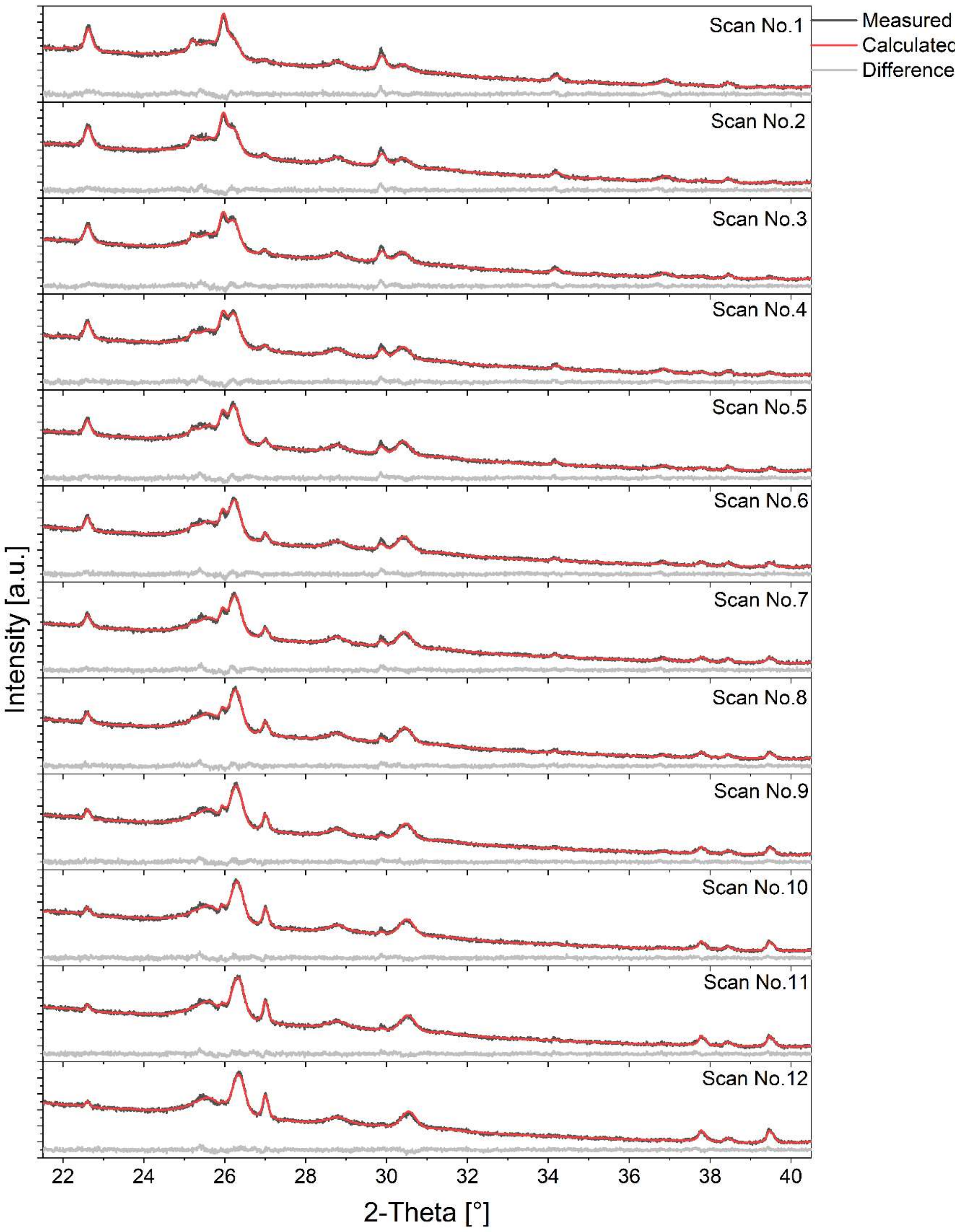

Figure S 10 Batch refinement of operando XRD patterns scan No.1-12 plot with fit and difference curve.

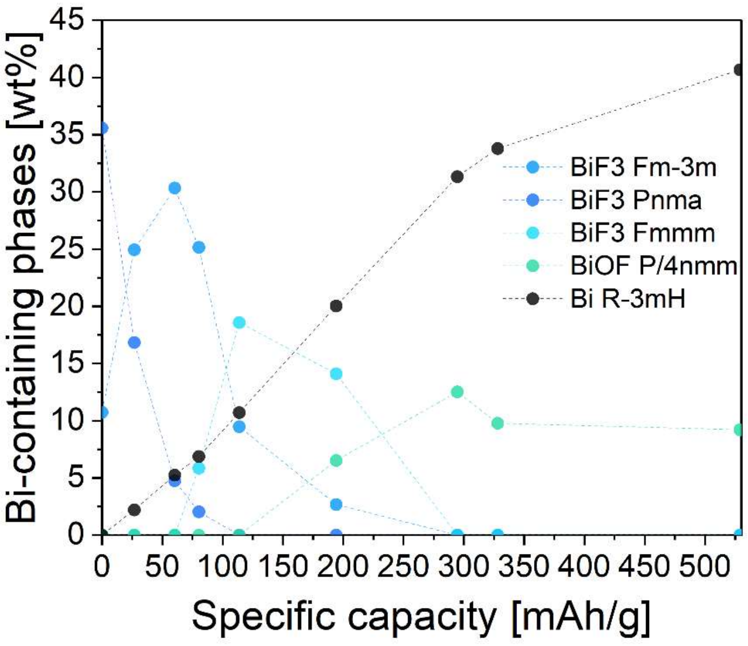

Figure S 11 Bi-containing phases obtained from manually refinement on selected operando XRD measurement scan No. 1, 5, 10, 13, 18, 30, 45, 50, 80, plotted as corresponding specific capacity.

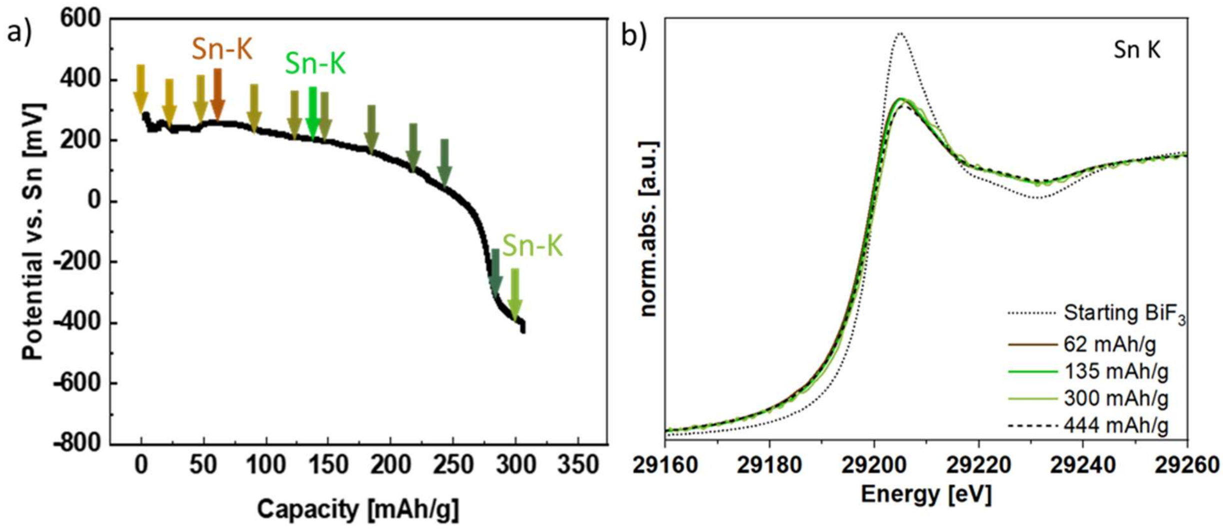

Figure S 12 a) cell potential against Sn/SnF$_2$ anode composite as a function of specific capacity investigated during operando XAS measurement; At the beginning of the measurement the cell potential had unignorable influence resulting from strong ground potential from synchrotron center (current leakage), the potential value gradually stabilized. And the delivered specific capacity as well as the XRD pattern of the cell after measurement both show the cell during operando XAS measurement behaves mostly similar to those investigated in ex-situ/operando XRD therefore exhibits high comparability. b) XAS spectra of Sn-K edge recorded at different specific capacity.

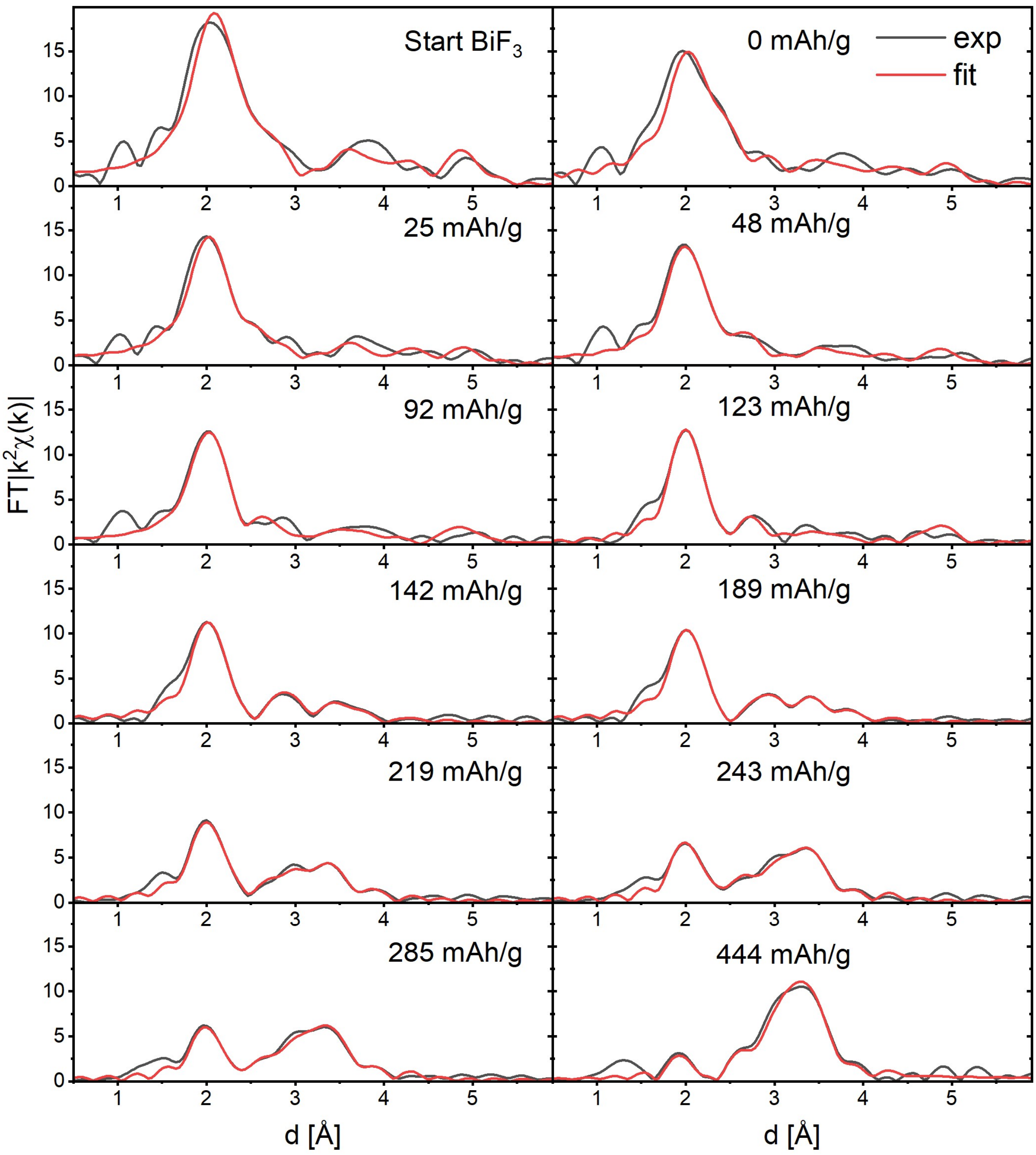

Figure S 13 Fourier transformed EXAFS spectra and the corresponding fits of the investigated samples.

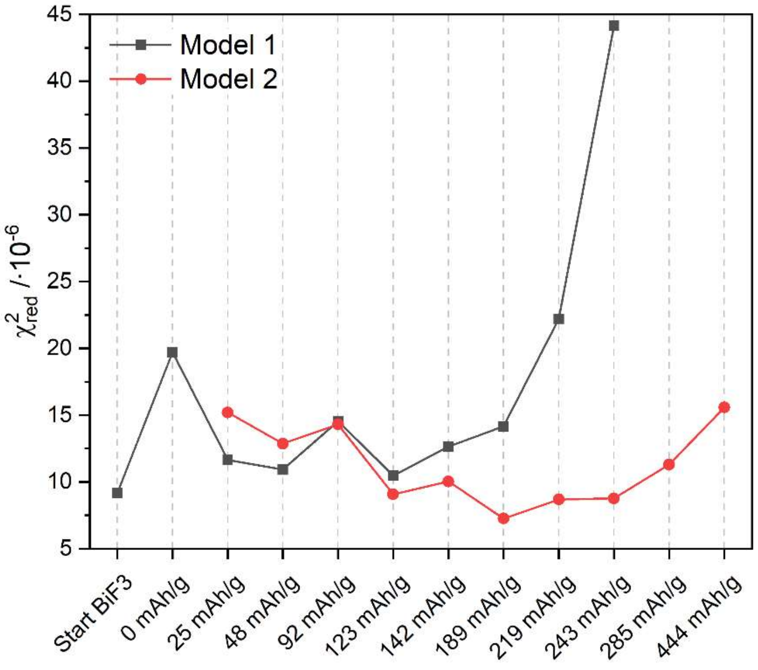

Figure S 14 Comparison of the χ^2_{red} fitting errors for the two adjusted models.

Table S 5 Unit cell volume per Bi in various Bi-containing phases from ICSD data base.

Bi-containing phase	Cell volume per Bi	Source of data
BiF_3 *Pnma*	55.75 Å^3	ICSD 1269
BiF_3 *Fm*-3*m*	49.98 Å^3	ICSD 24522
BiOF *P4/nmm*	43.71 Å^3	ICSD 24096
Bi *R*-3*m*	35.38 Å^3	ICSD 64703

Table S 6 Fitting results of the Fourier transformed spectra of the EXAFS region with two different models.

Sample	Bs	N (Bs)	R(Abs-Bs) [Å]	σ [Å^{-1}]	
	F	5.6 ± 0.6	2.274 ± 0.022	0.112 ± 0.011	R = 29.52 %
	F	1.9 ± 0.2	2.449 ± 0.024	0.067 ± 0.007	E_f = 10.34 eV
Starting BiF_3	F	0.6 ± 0.1	2.599 ± 0.026	0.032 ± 0.003	Afac = 0.8
	Bi	2.1 ± 0.2	3.873 ± 0.038	0.097 ± 0.010	χ^2_{red} = 9.18 ·10^{-6}
	Bi	0.3 ± 0.1	3.984 ± 0.039	0.032 ± 0.003	
	Bi	2.9 ± 0.3	4.439 ± 0.044	0.112 ± 0.011	
	F	3.5 ± 0.4	2.242 ± 0.022	0.102 ± 0.010	R = 36.05 %
	F	3.9 ± 0.4	2.452 ± 0.025	0.112 ± 0.011	E_f = 8.707 eV
0 mAh/g	F	0.5 ± 0.1	2.690 ± 0.027	0.045 ± 0.005	Afac = 0.8
Model 1	Bi	1.7 ± 0.2	3.859 ± 0.038	0.112 ± 0.011	χ^2_{red} = 19.71·10^{-6}
	Bi	0.6 ± 0.1	3.971 ± 0.040	0.063 ± 0.006	
	Bi	1.6 ± 0.2	4.464 ± 0.045	0.112 ± 0.011	
	F	1.3 ± 0.1	2.165 ± 0.022	0.077 ± 0.008	R = 30.51 %
	F	3.1 ± 0.3	2.314 ± 0.023	0.107 ± 0.011	E_f = 10.25 eV
25 mAh/g	F	1.5 ± 0.2	2.502 ± 0.025	0.112 ± 0.011	Afac = 0.8
Model 1	Bi	1.0 ± 0.1	3.860 ± 0.039	0.092 ± 0.009	χ^2_{red} = 11.65·10^{-6}
	Bi	0.2 ± 0.1	3.982 ± 0.040	0.032 ± 0.003	
	Bi	1.4 ± 0.1	4.430 ± 0.044	0.112 ± 0.011	
	F	0.5 ± 0.1	2.105 ± 0.021	0.032 ± 0.003	R = 29.77 %
	F	2.2 ± 0.2	2.250 ± 0.023	0.081 ± 0.008	E_f = 10.19 eV
48 mAh/g	F	2.2 ± 0.2	2.444 ± 0.024	0.112 ± 0.011	Afac = 0.8
Model 1	Bi	1.0 ± 0.1	3.843 ± 0.038	0.112 ± 0.011	χ^2_{red} = 10.92·10^{-6}
	Bi	0.2 ± 0.1	3.944 ± 0.039	0.059 ± 0.006	
	Bi	1.3 ± 0.1	4.419 ± 0.044	0.112 ± 0.011	
	F	0.4 ± 0.1	2.113 ± 0.021	0.055 ± 0.006	R = 33.51 %
	F	2.2 ± 0.2	2.239 ± 0.022	0.095 ± 0.010	E_f = 10.20 eV
92 mAh/g	F	1.7 ± 0.2	2.452 ± 0.025	0.110 ± 0.011	Afac = 0.8
Model 1	Bi	1.0 ± 0.1	3.861 ± 0.039	0.112 ± 0.011	χ^2_{red} = 14.55·10^{-6}
	Bi	0.2 ± 0.1	3.970 ± 0.040	0.112 ± 0.011	
	Bi	1.4 ± 0.1	4.429 ± 0.044	0.112 ± 0.011	
	F	0.6 ± 0.1	2.135 ± 0.021	0.032 ± 0.003	R = 31.97 %
	F	2.0 ± 0.2	2.286 ± 0.023	0.084 ± 0.008	E_f = 7.447 eV
123 mAh/g	F	1.7 ± 0.2	2.509 ± 0.025	0.112 ± 0.011	Afac = 0.8
Model 1	Bi	1.2 ± 0.1	3.859 ± 0.039	0.112 ± 0.011	χ^2_{red} = 10.48·10^{-6}
	Bi	0.3 ± 0.1	4.016 ± 0.040	0.063 ± 0.006	
	Bi	1.3 ± 0.1	4.452 ± 0.045	0.112 ± 0.011	
	F	1.1 ± 0.1	2.158 ± 0.022	0.071 ± 0.007	R = 29.33 %
	F	1.8 ± 0.2	2.342 ± 0.023	0.112 ± 0.011	E_f = 10.18 eV
142 mAh/g	F	0.5 ± 0.1	2.605 ± 0.026	0.077 ± 0.008	Afac = 0.8
Model 2	Bi	1.0 ± 0.1	3.056 ± 0.031	0.112 ± 0.011	χ^2_{red} = 10.04·10^{-6}
	Bi	0.1 ± 0.1	3.514 ± 0.035	0.045 ± 0.005	
	Bi	0.5 ± 0.1	3.686 ± 0.037	0.112 ± 0.011	
	F	1.0 ± 0.1	2.155 ± 0.022	0.067 ± 0.007	R = 26.35 %
	F	1.7 ± 0.2	2.336 ± 0.023	0.112 ± 0.011	E_f = 8.806 eV
189 mAh/g	F	1.0 ± 0.1	2.602 ± 0.026	0.107 ± 0.011	Afac = 0.8
Model 2	Bi	0.8 ± 0.1	3.054 ± 0.031	0.097 ± 0.010	χ^2_{red} = 7.26·10^{-6}
	Bi	0.3 ± 0.1	3.550 ± 0.036	0.081 ± 0.008	
	Bi	0.5 ± 0.1	3.741 ± 0.037	0.112 ± 0.011	
	F	0.8 ± 0.1	2.144 ± 0.021	0.067 ± 0.007	R = 28.89 %
	F	1.5 ± 0.2	2.318 ± 0.023	0.112 ± 0.011	E_f = 8.215 eV
219 mAh/g	F	1.0 ± 0.1	2.593 ± 0.026	0.112 ± 0.011	Afac = 0.8
Model 2	Bi	0.8 ± 0.1	3.052 ± 0.031	0.087 ± 0.009	χ^2_{red} = 8.69·10^{-6}
	Bi	0.6 ± 0.1	3.551 ± 0.036	0.105 ± 0.011	
	Bi	0.5 ± 0.1	3.751 ± 0.038	0.112 ± 0.011	
	F	0.4 ± 0.1	2.139 ± 0.021	0.050 ± 0.005	R = 29.56 %
	F	1.0 ± 0.1	2.310 ± 0.023	0.112 ± 0.011	E_f = 6.823 eV
243 mAh/g	F	0.9 ± 0.1	2.599 ± 0.026	0.112 ± 0.011	Afac = 0.8
Model 2	Bi	1.0 ± 0.1	3.055 ± 0.031	0.081 ± 0.008	χ^2_{red} = 8.77·10^{-6}
	Bi	0.7 ± 0.1	3.536 ± 0.035	0.112 ± 0.011	
	Bi	0.6 ± 0.1	3.721 ± 0.037	0.112 ± 0.011	
285 mAh/g	F	0.7 ± 0.1	2.158 ± 0.022	0.074 ± 0.007	R = 31.82 %
Model 2	F	1.1 ± 0.1	2.392 ± 0.024	0.112 ± 0.011	E_f = 6.047 eV
	F	0.7 ± 0.1	2.640 ± 0.026	0.084 ± 0.008	Afac = 0.8

	Bi	1.0 ± 0.1	3.055 ± 0.031	0.081 ± 0.008	χ^2_{red} = 11.31·10^{-6}
	Bi	0.8 ± 0.1	3.536 ± 0.035	0.112 ± 0.011	
	Bi	0.7 ± 0.1	3.729 ± 0.037	0.112 ± 0.011	
444 mAh/g	F	0.2 ± 0.1	2.145 ± 0.021	0.045 ± 0.005	R = 38.32 %
Model 2	F	0.7 ± 0.1	2.409 ± 0.024	0.112 ± 0.011	E_f = 4.285 eV
	F	0.4 ± 0.1	2.654 ± 0.027	0.039 ± 0.004	Afac = 0.8
	Bi	1.1 ± 0.1	3.056 ± 0.031	0.059 ± 0.006	χ^2_{red} = 15.57·10^{-6}
	Bi	0.3 ± 0.1	3.491 ± 0.035	0.032 ± 0.003	
	Bi	0.3 ± 0.1	3.655 ± 0.037	0.032 ± 0.003	

Bs = backscattering atom; N(Bs) = number of back-scattering atoms; R(Abs-Bs) = distance of absorbing to backscattering atom; σ = Debye–Waller-like factor; R = fit index; E_f = Fermi energy; Afac = amplitude reducing factor χ^2_{red} = reduced χ^2 fitting error

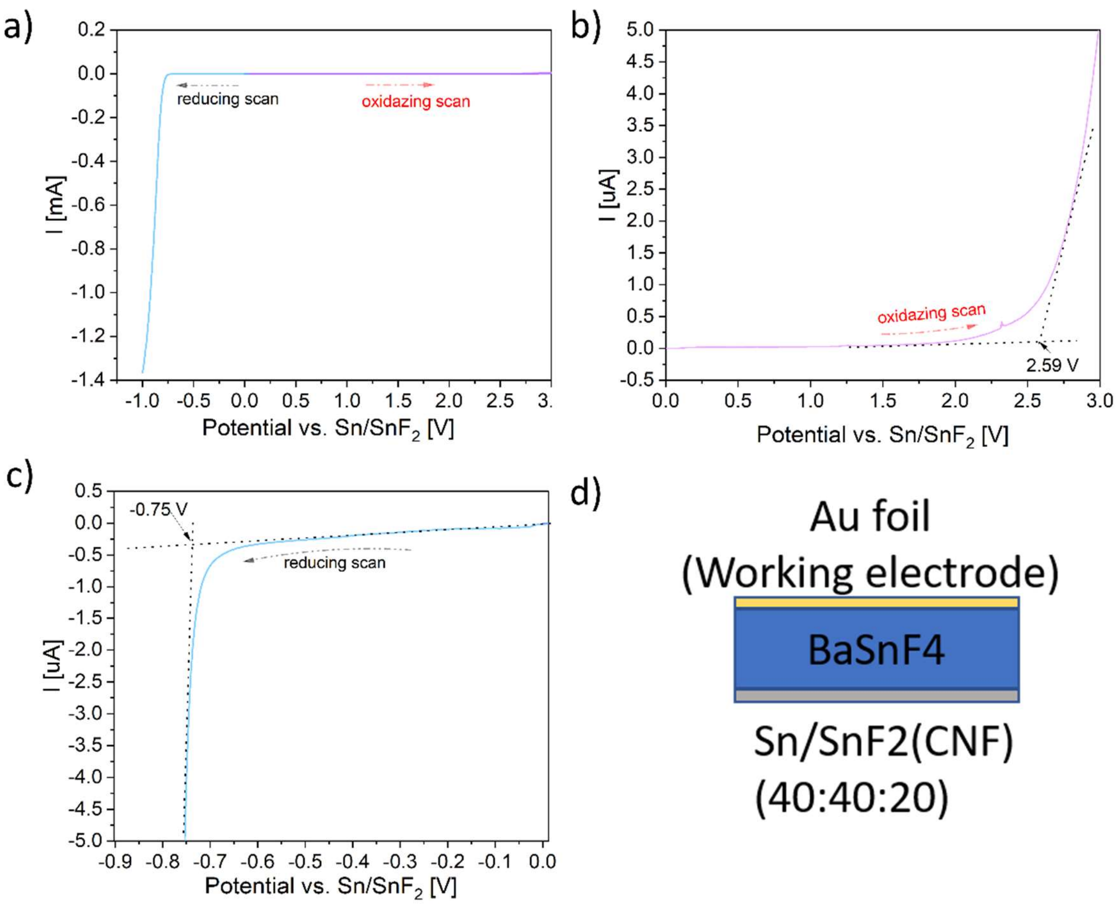

Figure S 15 The electrochemical stability window (ESW) of $BaSnF_4$ at 100 °C determined by LSV measurements. The cell configuration is demonstrated in d) and the scan rate is 0.1 mV s^{-1}.

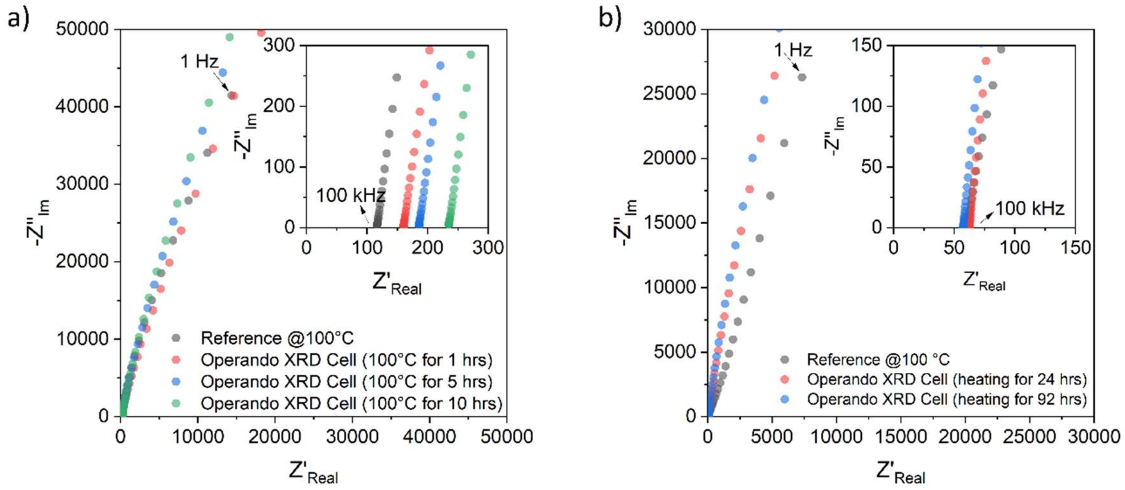

Figure S 16 Comparison of Impedance spectra for Au-coated electrolyte $BaSnF_4$ pellet measured at 100 °C in operando XRD cell under different sealing conditions: a) non-ideal sealing condition ($BaSnF_4$ batch A) and b) ideal sealing condition ($BaSnF_4$ batch B). Both $BaSnF_4$ batches exhibit identical structural characteristics in XRD patterns. Reference impedance spectra at 100 °C were obtained using MTZ CESH (Enhanced Controlled Environment Sample Holder) from BioLogic.

Comments on Figure S 16:

The negative influence of moisture from air on the electrolyte was observed, as evidenced by reduced ionic conductivity (which is sensitive for both surface and structure). The interaction between moisture and $BaSnF_4$ can be described as an acid-base reaction:

$BaSnF_4 + x\ H_2O \rightarrow BaSnF_{4-x}OH_x + x\ HF$

Figure S 16 demonstrates the impedance spectra of electrolyte at cell operating temperature (100 °C) under different sealing conditions. It is shown that with an improperly sealed cell, degradation of electrolyte (increased resistance) occurs within just 1 hr of heating. In contrast, our improved cell design (Figure 7 in Method section), which ensures proper sealing using optimized O-rings and lock washer, maintains stable electrolyte resistance even after 92 hrs of heating (Figure S 16b). This covers the required time duration for operando measurements. For Swagelok cell, sealing ferrules sit at the position far from the heating region (@RT) and therefore also maintains a good sealing condition. Based on the sealing test by EIS on our cells, we can reasonably rule out moisture absorption during electrochemical operation as oxygen origin. Given the elemental analysis results for all the precursors, electrolyte and composites (Line 171-183 and Table S 2 in SI in manuscript), we confirm the oxygen contributing to the formation of BiOF was introduced during preparation process into electrolyte and subsequently into composites. This rules out the relevance of external oxygen sources for our study, and the electrolyte oxygen impurities remain as the only plausible internal oxygen source in the system.
